# MemAF-KTCBM: a Memory-Aware Fractional optimisation-enabled deep learning model for atherosclerosis classification

**DOI:** 10.3389/fmedt.2026.1901028

**Published:** 2026-08-25

**Authors:** Nazarkar Pravalika, Jabeena A., Vetriveeran Rajamani

**Affiliations:** School of Electronics Engineering (SENSE), Vellore Institute of Technology, Vellore, Tamil Nadu, India

**Keywords:** angiographic images, atherosclerosis classification, deep learning, image augmentation, knowledge distillation

## Abstract

Atherosclerosis, a chronic vascular disorder caused by the abnormal functioning of blood vessels, remains a major contributor to cardiovascular diseases. Although recent advances in machine learning and deep learning have improved atherosclerosis classification, several critical challenges persist, particularly class imbalance, high computational complexity, limited interpretability, and poor generalisation, which constrain their clinical utility. To address these limitations, this paper proposes a novel Memory-Aware Fractional optimisation-assisted Knowledge-distilled mutual-conditioned dynamic Transformer-enabled Convolutional neural network-bidirectional long short-term Memory (MemAF-KTCBM) framework that employs knowledge distillation and mutual information fusion to reduce computational complexity while preserving classification performance. Initially, coronary artery structures are segmented using a memory-aware fractional optimisation-enabled Modified Pyramid Scene Parsing Network to improve classification reliability. Furthermore, a Memory-Aware Fractional optimisation-enabled Style Generative Adversarial Network is employed to generate high-quality synthetic angiographic images, thereby mitigating class imbalance and reducing prediction bias. In addition, pretrained local descriptors are incorporated to enhance feature extraction, interpretability, and classification reliability. The proposed framework is evaluated using the ARCADE and CADICA datasets using multiple training percentages and 10-fold cross-validation. On the ARCADE dataset, the MemAF-KTCBM model achieved an accuracy of 97.53%, a precision of 97.82%, a recall of 96.94%, an NPV of 0.97, a PPV of 0.98, and an MCC of 0.95. Similarly, on the CADICA dataset, it attained an accuracy of 96.65%, a precision of 96.94%, a recall of 96.07%, an NPV of 0.97, a PPV of 0.97, and an MCC of 0.94. These findings confirm that the proposed framework acts as a robust and effective solution for atherosclerosis classification across heterogeneous atherosclerosis datasets.

## 1 Introduction

Atherosclerosis refers to the category of ailments that mainly occur due to the malfunctioning of the pumping system of the heart, most specifically within the arteries (9). These diseases serve as the primary reason for mortality globally and are normally diagnosed on the basis of breathlessness, swollen feet, exhaustion, and physical weakness (10). Certain prime risk factors, including high blood pressure, smoking, sedentary lifestyles, and high cholesterol, are associated with this disease. Hence, early detection can aid in making effective treatment and save individual lives (11). Generally, atherosclerosis is a category of cardiovascular disease (CVD), and detection is performed by examining the risk factors such as family history, patient age, and lifestyle in the physical reports (12). These manual assessments result in erroneous predictions because they cannot capture the complex interplay among these risk factors, thereby increasing the computational complexity (13). Image processing modalities, such as magnetic resonance imaging (MRI), optical coherence tomography (OCT), coronary computed tomography angiography, and intravascular ultrasound, act as significant tools in the detection of heart diseases, providing high-resolution visualisations of the cardiac pathology and anatomy (14, 15).

Yet, extracting meaningful insights into atherosclerotic disease from these imaging modalities remains difficult, as it requires more robust image processing approaches to effectively handle large and complex datasets (16). Traditional methods have a crucial impact on the overall well-being of the individual (17). Although the deployment of artificial intelligence techniques in deep learning (DL) (34) and machine learning (ML) (35) methods can detect and classify atherosclerosis disease with significant results (18, 31), a lack of sufficient disease details and empirical data underscores the need for further exploration using ML techniques (19). In addition, certain challenges, such as class imbalance and a lack of interpretability, persist and hinder the practical application of conventional ML-based frameworks in healthcare (21). Furthermore, segmentation approaches, such as region growing, thresholding, active contour models, and edge detection, designed for effective atherosclerosis classification, are highly dependent on intensity gradients and predefined features, which reduces their robustness to noisy, complex imaging data (20). Even though transformer-based frameworks are designed for effective segmentation of cardiac regions in medical imaging modalities, their substantial requirement for additional computational resources and extensive data limits their use in clinical practice (22). Meanwhile, the DL models acquire deeper features through their highly interconnected neural networks, producing reliable findings (23). DL models, such as CNNs, deep belief networks, and recurrent neural networks (RNNs), are deployed for processing medical images (24). Yet, the prevalence of overfitting issues, vanishing-gradient challenges, and huge data requirements increased the need for efficient models (25). Moreover, hybrid models are designed with long short-term memory (LSTM) and RNN architectures for improved disease diagnosis but with reduced generalisation, thereby increasing computational complexity (26).

Therefore, this paper describes an effective MemAF-KTCBM model to address existing limitations in atherosclerosis classification. Extracting important features using the pretrained local descriptor improves atherosclerosis classification. Eventually, the application of StyleGAN-based image augmentation and the MSPSNet-based segmentation process reduces computational complexity while extracting important features. In particular, the use of standardised input medical images improves the accuracy of atherosclerosis classification by highlighting significant atherosclerosis-related features. Some of the major contributions of the MemAF-KTCBM are discussed as follows.

Memory-Aware Fractional (MemAF) optimisation algorithm: The MemAF optimisation algorithm integrates the higher sensing characteristics of the Sand Cat into the serval approach through the effective use of fractional calculus. This mechanism improves optimal search performance by eliminating premature convergence issues, leading to better convergence with less computation time.

MemAF-based Knowledge-distilled mutual-conditioned dynamic Transformer-enabled Convolutional neural network-Bidirectional long short-term Memory (MemAF-KTCBM) model: The MemAF-KTCBM model contributes to accurate classification performance by aggregating finer details using knowledge distillation, employing a teacher and student model to extract critical disease features. The teacher model incorporates a CNN, vision transformer (ViT), and BiLSTM network to learn important features and predict disease uncertainties. Consequently, the student model imitates the learning from the teacher model to achieve accurate atherosclerosis classification with reduced loss via the knowledge distillation mechanism.

The structural arrangement of the paper is as follows: Section 2 reviews the traditional works on atherosclerosis classification. Section 3 details the MemAF-KTCBM-based atherosclerosis classification methodology. The experimental verification of the MemAF-KTCBM is given in Section 4, followed by the conclusion in Section 5 with future extensions.

## 2 Literature review

Recent studies on atherosclerosis and cardiovascular disease classification were reviewed to identify the strengths and limitations of existing approaches. The studies summarised in Table 1 were selected based on their relevance to coronary image analysis, segmentation, feature extraction, optimisation, and deep learning-based classification techniques that are closely related to the proposed MemAF-KTCBM framework. The identified limitations were the primary motivation for developing the proposed methodology.

**Table 1:** Summary of state-of-the-art methods for atherosclerosis classification and their associated limitations.

Author(s)	Method	Key contribution	Limitation
Zhao et al. (1)	Multi-stream Attention-guided Coronary Analysis Network	Combined localisation and segmentation through dual-stream architecture with spatial and channel attention modules for effective feature extraction	Inability to maintain consistent classification across different phases and reduced detection performance
Huang et al. (2)	Segment Anything Model-Vision Mamba-UNet	Utilised self-attention and long-range dependency learning for improved disease detection	Lossy characteristics resulted in the loss of local and structural features, reducing generalisability
Al Gharib et al. (3)	Hybrid Polynomial Ensemble Model	Improved detection accuracy and interpretability through polynomial feature extraction and mutual information-based feature selection	Higher false-positive rates indicated a trade-off between sensitivity and specificity
Zhang et al. (4)	Context-Interactive Deep Network	Integrated multi-scale interactive and bio-inspired attention blocks for effective edge and spatial feature extraction	Susceptible to overfitting, leading to unreliable detection performance
Deng et al. (5)	Dual Multi-Scale Feature Aggregation Network	Enhanced multi-scale feature learning and active coronary vessel segmentation	Limited data availability and large training data requirements reduced reliability
Khan et al. (6)	Ensemble and Blending-based Network	Employed Shapley Additive Explanations for feature contribution analysis and heart disease classification	Extensive preprocessing increased computational resource requirements and processing time
Elsedimy et al. (7)	QPSO-enabled support vector machine (SVM)	Optimised SVM parameters using adaptive thresholding to avoid local minima	Data imbalance, overfitting, and limited scalability affected performance
Vinay et al. (8)	GAN-assisted ML gramework with RNN-BiLSTM	Generated high-quality synthetic data to mitigate class imbalance and improve detection accuracy	Reduced generalisability and difficulty in capturing long-range dependencies increased computational complexity

To address the above-mentioned limitations, the proposed MemAF-KTCBM framework comprise several distinct components, each designed to provide a specific benefit. First, an advanced preprocessing strategy is employed to enhance image quality, improving the reliability of subsequent analysis. Second, the MemAF-SGAN-based augmentation mechanism is introduced to alleviate class imbalance and reduce overfitting, thereby improving model robustness. Third, the MPSPNet-based segmentation module accurately delineates coronary artery structures, enabling more precise identification of relevant anatomical regions. In addition, multiple feature extraction techniques are used to capture discriminative characteristics, enhancing the representation of disease-related patterns. Finally, a knowledge distillation mechanism is incorporated to reduce computational complexity and minimise classification loss, improving efficiency without sacrificing performance. Collectively, these components enable robust and accurate atherosclerosis classification while enhancing the generalisation capability of the model.

### 2.1 Problem statement

Atherosclerosis is a condition that normally occurs when the normal functioning of the heart is disrupted by plaque formation in the arteries. Even though ML models have brought a major transformation in the healthcare industry, the presence of imbalance issues resulted in biased predictions (1, 5). However, the single-dataset dependence of the existing ML models reduced their generalisability (3). Fortunately, the development of DL models with numerous hidden layers has enabled accurate identification of complex features and improved classification performance. Conversely, the large data requirement for atherosclerosis classification increased computational complexity (4) with additional resource requirements (6). Therefore, this study introduces an effective MemAF-KTCBM to perform accurate atherosclerosis classification using x-ray angiographic images. The MemAF-KTCBM-based atherosclerosis classification is envisaged as follows.

The x-ray angiographic images are used as initial input from the automatic region-based coronary artery disease (ARCADE) and CADICA datasets to perform atherosclerosis classification via the MemAF-KTCBM model. The mathematical representation of the x-ray angiographic images acquired from these two specific datasets is expressed in Equation 1

$$D=\{X_{i},Y_{i}\},1\leq i\leq n$$ (1)

where $X_{i}$ represents the ith input x-ray angiographic image present in the dataset *D* and is annotated as $X_{i}\in {ℜ}^{a\times b\times c}$, a×b×c indicates the height, width, and channel of the ith x-ray angiographic image, $Y_{i}$ signifies the labels corresponding to the ith x-ray angiographic image, and *n* represents the total number of images present in *D*.

Initially, the input images are preprocessed to produce high-quality images and fed into the MemAF-SGAN-based augmentation to generate new samples with better generalisability. Thus, the newly generated sample *x* using the MemAF-SGAN model is expressed in Equation 2 as follows:

x=GAN(z,θ) (2)

where *z* refers to the latent vector from the Gaussian prior and is represented as z∼z(0,1), θ denotes the learnable parameter of *GAN*. For each class present in the dataset, the generation of synthetic samples $\left({\hat{x}}_{k}^{c}\right)$ takes place, expressed in Equation 3 as follows:

$${\hat{x}}_{k}^{c}=GAN(z_{k}^{c}),1\leq k\leq \max\mathrm{count}$$ (3)

Here, *k* indicates the maximum number of generated samples. Let the labels after augmentation be ${\hat{y}}_{k}^{c}=c$, where *c* unveils the labels of *D*. The obtained augmented data $\bar{D}$ is modelled using the following Equation 4:

$$\bar{D}=D\cup \{\left({\hat{x}}_{k}^{c},{\hat{y}}_{k}^{c}\right)\}$$ (4)

where $\left({\hat{x}}_{k}^{c}\right)$ represents the synthetic samples and ${\hat{y}}_{k}^{c}$ represents the augmented labels. The use of the Modified Pyramid Scene Parsing Network (MPSPNet) aids in accurate segmentation through its multi-scale contextual information aggregation. The pretrained local descriptor extracts information-rich features. Conceptually, MemAF-KTCBM is a student–teacher model whose parameters are optimised using the MemAF optimisation algorithm to achieve optimal atherosclerosis classification performance with reduced loss.

The output labels of the ARCADE dataset are detailed as follows:

$$\begin{array}{rl} Y_{i}\in \; & 0,1,2,3,4,5,6,7,8,9,9a,10,10a,11,12,12a,13,\\ \; & 14,14a,15,16,16a,16b,16c,12b,14b \end{array}$$ (5)

where 1 denotes the right coronary artery (RCA); 2 is the right coronary artery (RCA) mid; 3 is the restricted cubic spline (RCS) distal; 4 denotes the posterior descending artery; 5 represents the left main; 6 is the left anterior descending (LAD) artery proximal; 7 is the LAD mid; 8 is the LAD apical; 9 and 9a are the first diagonal branch and the additional first diagonal branch, respectively; 10 and 10a are the second diagonal and the additional second diagonal branch, respectively; 11 models the proximal circumflex artery; 12 models the intermediate/anterolateral artery; 12a and 12b represent the first obtuse marginal branch and the second obtuse marginal branch, respectively; 13 represents distal circumflex, proximal; 14, 14a, and 14b are the left posterolateral branch (first), second left posterolateral branch, and third left posterolateral branch, respectively; 15 represents posterior descending; and 16, 16a, 16b, and 16c indicate posterolateral branches from the RCA.

The output labels of the CADICA dataset are detailed in the following Equation 6:

$$Y_{i}\in \{\begin{array}{cc} 0, & \mathrm{Non}\text{-}\mathrm{ST}\;\mathrm{segment}\;\mathrm{elevation}\;\mathrm{acute}\;\mathrm{coronary}\;\mathrm{syndrome}\\ 1, & \mathrm{ST}\;\mathrm{segment}\;\mathrm{elevation}\;\mathrm{acute}\;\mathrm{coronary}\;\mathrm{syndrome}\\ 2, & \mathrm{Stable}\;\mathrm{angina} \end{array}$$ (6)

The atherosclerosis classification performance of the MemAF-KTCBM is optimised via the knowledge distillation loss $\left(X_{loss}\right)$, as depicted in Equation 7. $X_{loss}$ is the linear combination of $X_{cce}$ and $X_{kld}$. Here, $X_{cce}$ is the categorical cross-entropy loss, and $X_{kld}$ indicates the Kullback–Leibler divergence loss:

$$X_{loss}=(1-\alpha_{1})X_{cce}(\;p^{s}(1),y)+\alpha_{1}X_{kld}(\;p^{s}(\tau ),p^{t}(\tau ))$$ (7)

In Equation 8, $y_{i}$ denotes the ground-truth one-hot encoded label corresponding to class *j*, while $p_{j}^{s}$(1) represents the probability predicted by the student network for class *j* at temperature 1:

$$X_{cce}(\;p^{s}(1),y)=\sum_{j}-y_{j}\log p_{j}^{s}(1)$$ (8)

$$X_{kld}(\;p^{s}(\tau ),p^{t}(\tau ))=\tau^{2}\sum_{j}\;p_{j}^{t}(\tau )\log\frac{\;p_{j}^{t}(\tau )}{\;p_{j}^{s}(\tau )}$$ (9)

In Equation 9, *t* represents the teacher network, τ denotes the temperature scaling hyperparameter, *s* denotes the student network, *y* refers to the one-hot label vector of the xth sample, and $\alpha_{1}$ indicates the linear combination hyperparameter.

Collectively, the deep learning architecture of MemAF-KTCBM effectively analysed medical image features and disease characteristics to improve atherosclerosis classification accuracy. Furthermore, segmenting only the cardiac structures with the MPSPNet reduced the additional parameter requirements for aggregating multi-scale contextual details. Generating synthetic samples using MemAF-SGAN eliminated data imbalance issues and improved generalisability. Furthermore, active tuning using the MemAF eliminates premature convergence issues and improves the model stability.

## 3 Proposed memory-aware fractional optimisation-enabled deep learning model for cardiovascular disease classification

**Figure 1:**
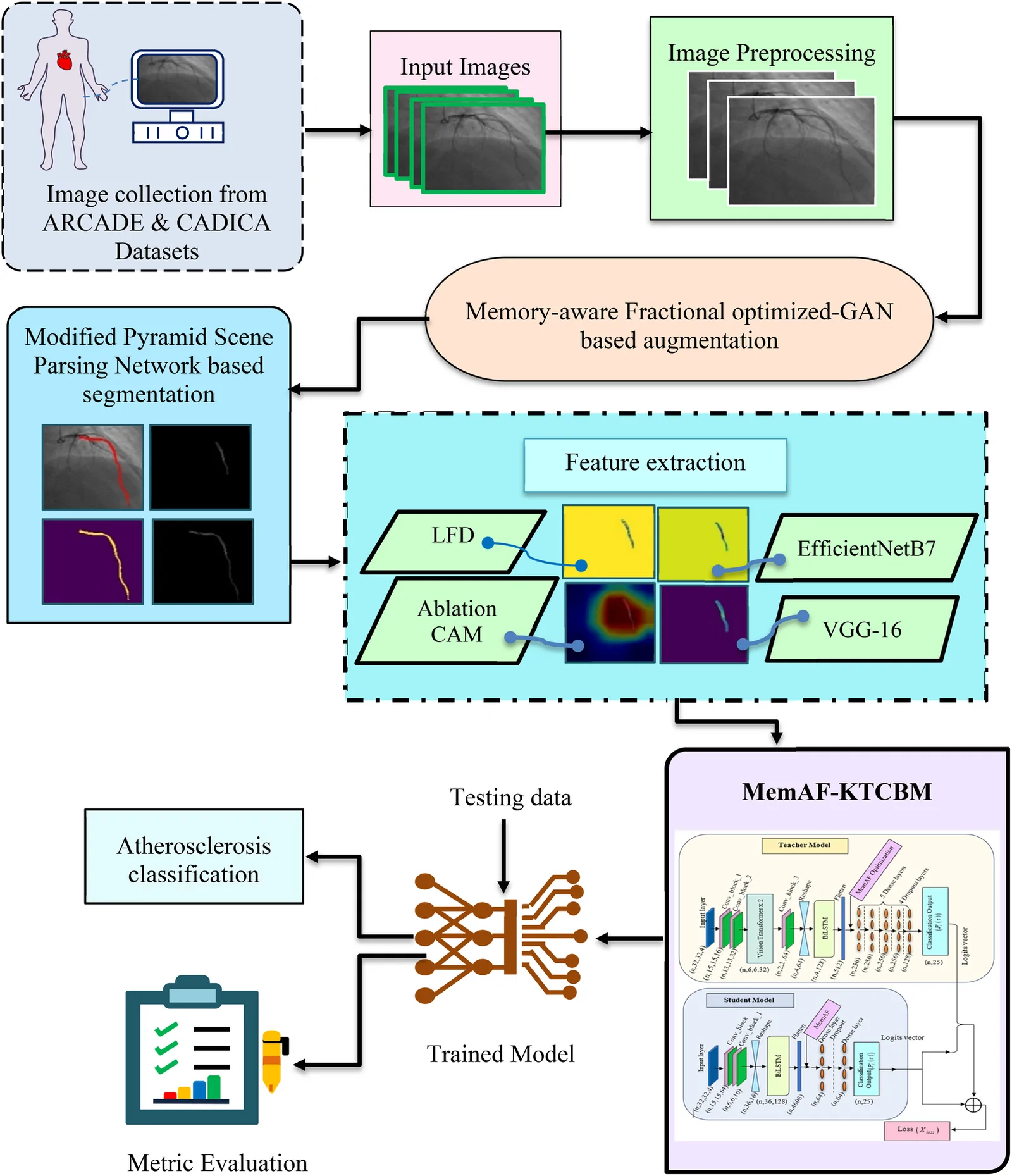
Schematic illustration of the MemAF-KTCBM model for atherosclerosis classification.

The proposed methodology is an effective MemAF-KTCBM model for classifying atherosclerotic disease by analysing the input medical image. In the atherosclerosis classification process, MemAF-KTCBM initially sources the input image from the ARCADE and CADICA datasets. Primarily, the input image undergoes gamma correction approaches to provide detailed visibility of the cardiac disease-related abnormalities by improving image contrast. Afterward, high-quality synthetic images are generated using MemAF-SGAN to eliminate data scarcity issues in atherosclerosis classification. The generated synthetic image samples are resized and converted to greyscale for accurate segmentation using MPSPNet. In particular, MPSPNet aids in the segmentation of complex cardiac structures, achieving higher segmentation accuracy to improve atherosclerosis classification performance and reduce computational overhead. Then, informative feature extraction is performed to reduce additional resource requirements and speed up the atherosclerosis classification process. The extracted features are then input to MemAF-KTCBM for accurate atherosclerosis classification. Nevertheless, the MemAF-KTCBM parameters are fine-tuned using the MemAF optimisation algorithm to achieve optimal classification performance by eliminating issues related to premature convergence. The MemAF-KTCBM model is schematically illustrated in Figure 1.

### 3.1 Input medical images

In this research, input x-ray angiographic images are sourced from the ARCADE (29) and CADICA (30) datasets with dimensions (n,512,512,3) to perform atherosclerosis classification. The mathematical annotation for the input medical image is given in Equation 1, which undergoes quality enhancement via preprocessing in the subsequent step to accurately classify atherosclerosis.

### 3.2 Medical image preprocessing

The input image $X_{i}$ with dimension (n,512,512,3) is preprocessed, facilitating accurate atherosclerosis classification by improving the crucial image attributes linked with atherosclerosis. Conceptually, the research uses the preprocessing technique known as gamma correction to enhance medical image quality in a non-linear manner by adjusting image brightness. Fortunately, gamma correction ensures perceptual uniformity, data compression, and display consistency. Gamma correction is applied to the input image with intensity *I* and is mathematically formulated in the following Equation 10:

$$X_{i}(I)=I^{\varsigma }$$ (10)

where I∈[0,1] is the image pixel intensity and ς indicates the gamma value and is found to be 1.5 for this research. Hence, the quality-enhanced preprocessed image with dimension (n,512,512,3) is denoted $X_{i}^{*}$, which is further provided to MemAF-SGAN for medical image augmentation.

### 3.3 Medical image augmentation using Memory-Aware Fractional optimised-Style Generative Adversarial Network (MemAF-SGAN)

**Figure 2:**
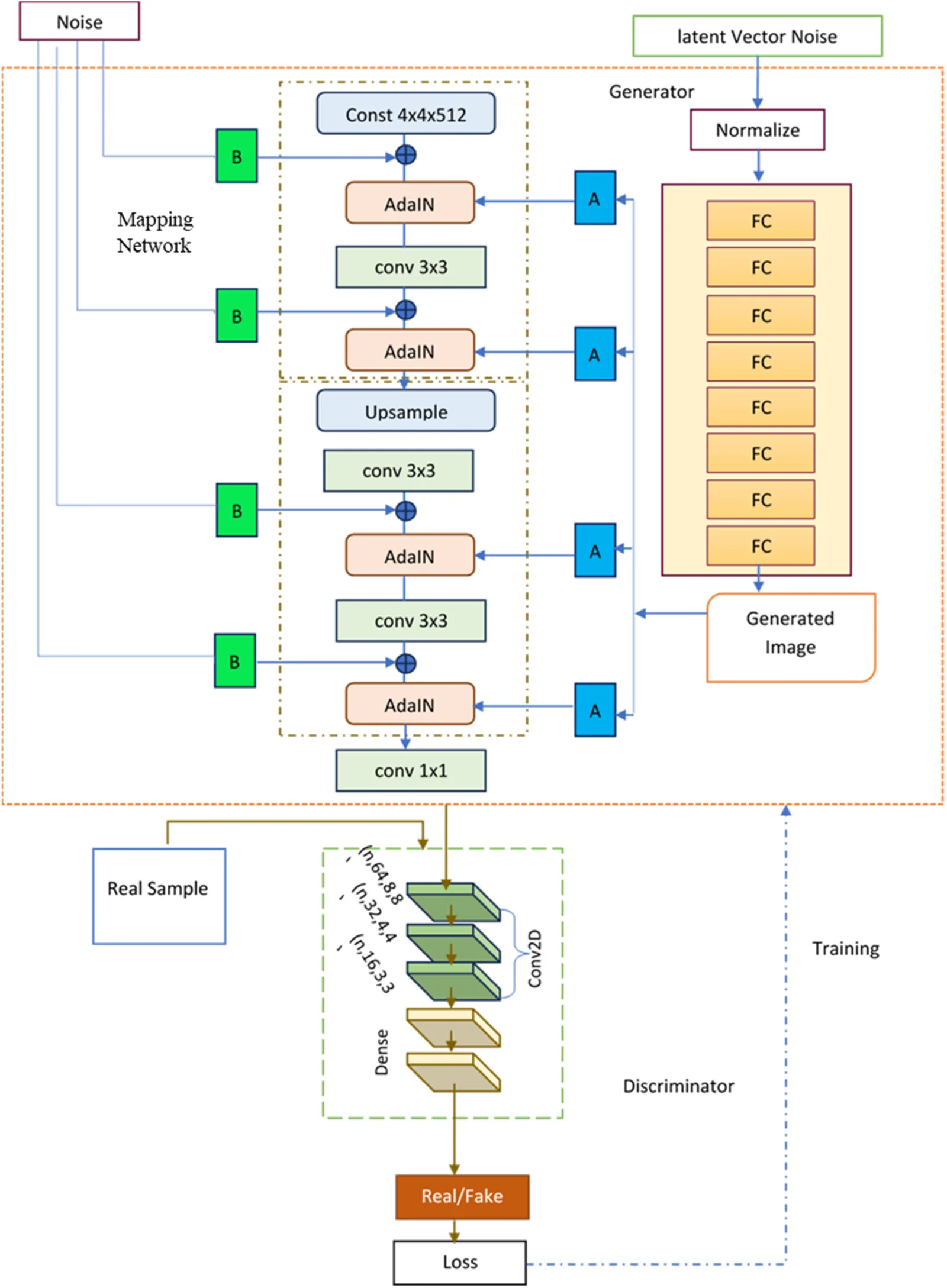
Architecture of MemAF-SGAN.

The preprocessed image $X_{i}^{*}$ with dimension (n,512,512,3) is forwarded to the image augmentation technique, where the MemAF optimisation-based StyleGAN is used to generate diverse, highly correlated synthetic images to reduce overfitting by eliminating class imbalance. Here, the StyleGAN generates high-resolution characteristic images rather than producing copies of input images for training data. Hence, the MemAF-SGAN for the medical image augmentation process improves its generalisation characteristics to new, unseen data and helps improve the overall performance. Moreover, the application of MemAF optimisation fine-tunes the weight parameters of the GAN model to achieve higher accuracy and fewer errors. MemAF-SGAN (16) comprises three networks: the mapping network, the generator $\left(\bar{G}\right)$, and the discriminator $\left(\bar{D}\right)$, as shown in the detailed architecture in Figure 2.

The training process of MemAF-SGAN generally reflects the fooling nature of the generator and the accurate classification of real and fake samples by the discriminator. The formula governing this image augmentation process is given in the following Equation 11:

$$\underset{\bar{G}}{\min}\underset{\bar{D}}{\max}\;V(\bar{D},\bar{G})=E_{X_{i}^{*}∼P_{data}(X_{i}^{*})}[\log(\bar{D}(X_{i}^{*}))]+E_{z∼P_{z}(z)}[\log(1-\bar{D}(\bar{G}(z,\psi )))]$$ (11)

where $V(\bar{D},\bar{G})$ represents the objective function, $X_{i}^{*}∼P_{data}(X_{i}^{*})$ refers to the samples obtained from real data distribution, $z∼P_{z}(z)$ denotes the noise vector sampled from an *a priori* distribution, and $\bar{D}(X_{i}^{*})$ and $\bar{D}(\bar{G}(z))$ indicate the discriminator predictions on real and fake image samples, respectively. The mapper network is an eight-layer neural network that takes random noise, denoted *z*, of dimension (512,512,3) as its input and transforms it into a style signal or an intermediate latent vector (ψ). Here, the latent vector defines the style of medial image generation. The image style thus generated in the form of ψ then flows into the generative network, which uses the Adaptive Instance Normalisation (AdaIN) technique to produce an image with good control over its style at each resolution. Moreover, along with ψ, the generator network receives *z* as input and adds it after each convolution layer to compute non-linearity. The generator network comprises 18 layers to generate synthetic images. Specifically, the image generated at the last layer of the generator undergoes red green blue (RGB) conversion via convolution with a kernel size of 1×1. For better control of image styles, the AdaIN technique converts ψ into two distinct scalars or styles (ζ), denoted scale $\left(\zeta_{sc}\right)$ and bias $\left(\zeta_{bi}\right)$ in (12), using learned affine transformations, shown in Equation 12 as follows:

$$\zeta =(\zeta_{sc},\zeta_{bi})$$ (12)

The AdaIN function is formulated in Equation 13 as follows:

$$AdaIN(X_{i}^{*},\zeta )=\zeta_{sc}\frac{X_{i}^{*}-\beta_{1}(X_{i}^{*})}{\beta_{2}(X_{i}^{*})}+\zeta_{bi}$$ (13)

where $\beta_{1}$ and $\beta_{2}$ represent the mean and standard deviation of $X_{i}^{*}$, respectively.

The fake image samples thus generated at the generator network are modelled as $\bar{G}(z,\psi )=x^{\prime }$. Eventually, the samples thus generated at $\bar{G}$ are closer to the data distribution of $X_{i}^{*}$, indicated as $P_{data}(X_{i}^{*})$. Simultaneously, the discriminator $\left(\bar{D}\right)$ is provided with the generated fake samples $\left(x^{\prime }\right)$ to compute the predicted output in the interval [0,1]. In addition, $\bar{D}$ also receives input to determine the predicted output for the same interval. The input $\left(A\in \{X_{i}^{*},x^{\prime }\}\right)$ thus received at the discriminator with dimension (n,512,512,3) firstly enters the input layer of the discriminator and is processed effectively in the successive two-dimensional (2D) convolutional layer to acquire the relevant features of dimension (n,64,8,8) by performing a convolution operation, given in Equation 14 as follows:

$$X_{conv}^{*}(a,b)=\sum_{a}\sum_{b}A[a,b]\cdot \hbar (r-a,s-b)$$ (14)

where ℏ(r,s) denotes the impulse response and [a,b]- represents the pixel coordinates.

In addition, to optimise the weight and bias values of MemAF-SGAN, the research uses the MemAF optimisation algorithm. These relevant features then propagate through two 2D convolutional layers and a dense layer with a rectified linear unit (ReLU) and sigmoid activation functions to provide the output $\left(S_{out}\right)$ as either real or fake with dimension (n,1) by deeply analysing the extracted relevant features. Finally, backpropagation adjusts the weights of these three networks to generate high-quality images in successive iterations. Thus, the output obtained as $S_{gan}$ after medical image augmentation using MemAF-SGAN has a dimension of (n,512,512,3), indicating an increase in the number of samples.

Since the dimension of the augmented image is quite high, it becomes very difficult to segment the cardiac structures to perform accurate classification. Hence, $S_{gan}$ undergoes resizing followed by grayscale conversion before entering the segmentation module for effective processing. Thus, the augmentation output is generated as a greyscale image, denoted $S_{gry}^{*}$, with dimension (n,256,256,3).

### 3.4 Medical image segmentation using the Modified Pyramid Scene Parsing Network

**Figure 3:**
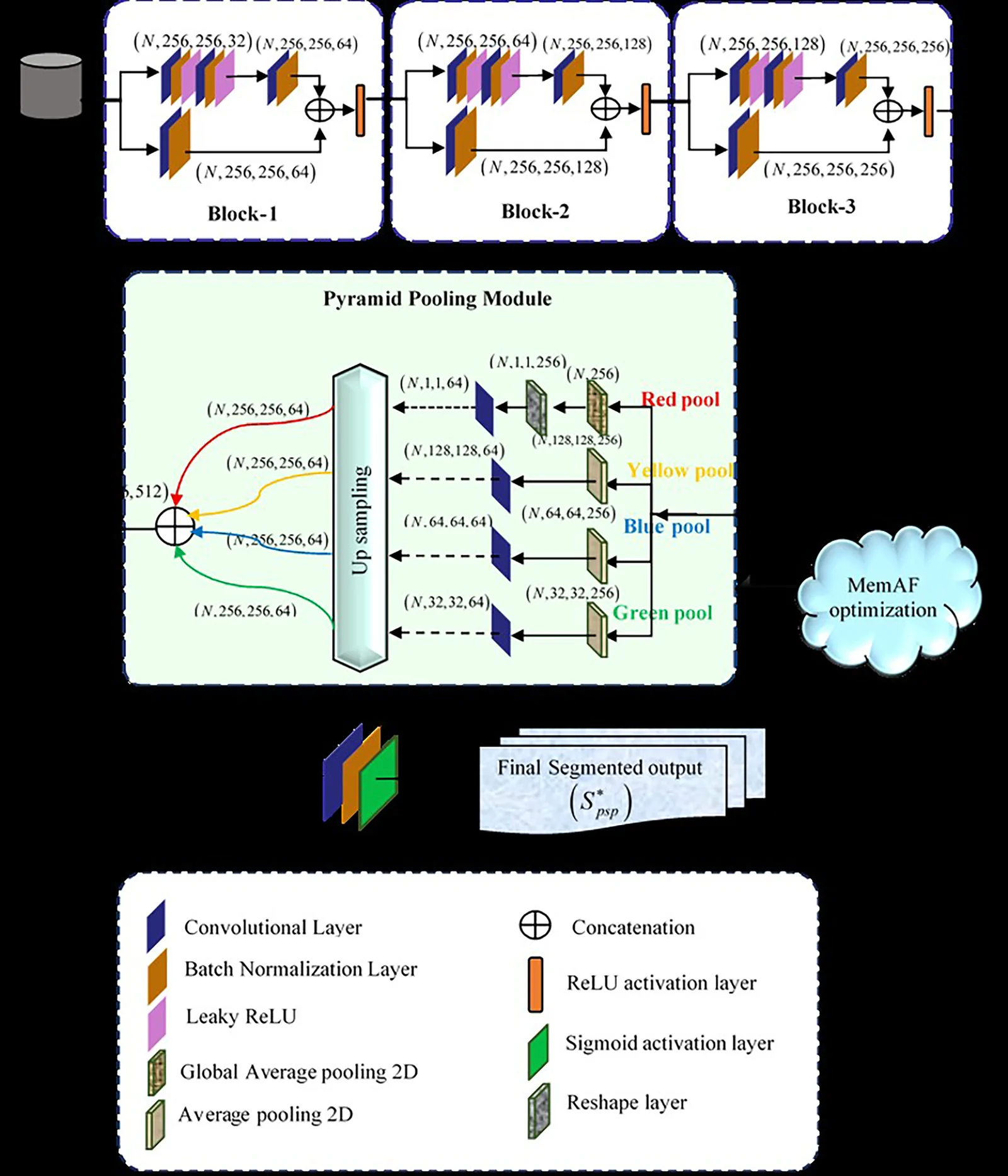
Architecture of MPSPNet-based segmentation.

The augmented image $S_{gry}^{*}$ with dimension (n,256,256,3) is applied to the segmentation process, where MPSPNet is employed to capture contextual features over diverse scales. The MPSPNet-based image segmentation (17) facilitates accurate image segmentation by aggregating diverse details using its pyramid pooling module. The MPSPNet encompasses an input layer, three convolutional blocks with convolutional layers, batch normalisation, a leaky ReLU layer, and a ReLU activation layer in its architecture to perform segmentation tasks. As illustrated in Figure 3, each convolutional block divides the input into two distinct branches. Branch 1 contains two different sets of 2D convolutional layers, a leaky ReLU layer, and batch normalisation, while Branch 2 is equipped with a singular configuration of 2D convolutional layers accompanied by a batch normalisation layer. The outputs from both branches are combined to generate the complex features with dimension (n,256,256,256) and sent to the pyramid pooling module. The intricate components integrated into the pyramid pooling module are subjected to scaling across four distinct pooling pyramids: red pool, yellow pool, blue pool, and green pool, to extract the global contextual features. The global contextual features are instrumental in defining the categories of atherosclerosis. Within the four distinct pooling pyramids, the red pool implements a global average pooling 2D strategy, while the remaining three pools utilise the average pooling strategy to extract complex semantic features. The MemAF optimisation algorithm is implemented before the pyramid pooling module in the MPSPNet to refine the model parameters. The complex features extracted from these four diverse pools are then concatenated and passed through a 2D convolutional layer, a batch normalisation layer, and a sigmoid activation layer to generate the segmented image $\left(S_{\;psp}^{*}\right)$ with dimensions (n,256,256,1). The segmented output then undergoes effective feature extraction in the subsequent stage to facilitate accurate atherosclerosis classification. Figure 3 explains the working flow of the MPSPNet-based segmentation process.

### 3.5 Feature extraction using the pretrained local descriptor

The feature extraction module receives the segmented image $\left(S_{\;psp}^{*}\right)$ with dimension (n,256,256,1) as input to identify highly associated atherosclerosis discriminant features, thereby decreasing duplicate features and resource needs (33). The feature extraction process incorporated the pretrained local descriptor to correlate the characteristics of LFD, VGG16, EfficientNet B7, and Ablation CAM. Further details on extracting such critical traits are provided.

#### 3.5.1 Local frequency descriptor features

The LFD features (22) explore both the magnitude and phase details of the wider frequency band features from $S_{\;psp}^{*}$ to assess the effectiveness of atherosclerosis classification even in low-resolution images. To capture both the magnitude information and the relative relationships among distinct patches in $S_{\;psp}^{*}$, the blur-invariant property of the descriptor is exploited. The formula for magnitude computation $\left(\;f_{mag}\right)$ is given in Equation 15 as follows:

$$f_{mag}[a,b]=\sum_{\hat{k}=1}^{8}\hat{H}(S_{\;psp}^{*})2^{\hat{k}-1}$$ (15)

where $\hat{k}=1,2,3,\ldots ,8$ represents the pixels in $S_{\;psp}^{*}$ and $\hat{H}(S_{\;psp}^{*})$ indicates the relationship between the neighbouring patches. Likewise, the phase information $\left(\;f_{\;phs}\right)$ is computed as given in Equation 16:

$$f_{\;phs}[a,b]=\sum_{\hat{k}=1}^{8}\hat{G}(S_{\;psp}^{*})2^{\hat{k}-1}$$ (16)

where $\hat{G}(S_{\;psp}^{*})$ indicates the relative phase details with distinct patches. The combination of both $f_{mag}$ and $f_{\;phs}$ generates the LFD features of dimension (n,256,256,1). The resulting wider frequency band features are then resized (n,32,32) to improve the accuracy of atherosclerosis classification by reducing the training and classification time and are denoted $f_{lfd}$.

#### 3.5.2 Visual Geometry Group 16 (VGG-16) features

VGG-16 (21) extracts the spatial features accurately using its 16-layer architecture, excluding the pooling layers. VGG-16 comprises 13 convolution layers, 3 fully connected layers, and 5 max-pooling layers. Each of these layers is organised into six main stages, and spatial feature extraction takes place at the output of the third layer. These spatial features are found to have the dimension of (n,112,112,64). The dimension of these spatial features is further reduced and made suitable for easy processing in MemAF-KTCBM through resizing. Hence, the resulting VGG-16-based spatial feature obtained after resizing to the dimension (n,32,32) is modeled as $f_{vgg16}$.

#### 3.5.3 Ablation class activation Map (Ablation-CAM) features

Ablation-CAM (20) provides better visualisation and extraction of the fine-grained features from $S_{\;psp}^{*}$. To do so, Ablation-CAM analyses the weights $\left(w_{\hat{k}}\right)$ of each feature across its diverse classes and removes the specific image feature. After removing the specific image feature, Ablation-CAM examines whether the image characteristics remain the same. In addition, Ablation-CAM generates a high-quality coarse localisation map, providing better visualisation of accurate atherosclerosis classification. The formula for Ablation-CAM feature extraction $\left(\;f_{abla}\right)$ is given in Equation 17 as follows:

$$f_{abla}=℘\left(\sum_{\hat{k}}\omega_{\hat{k}}\cdot S_{\;prp}^{*}\right)$$ (17)

where ℘ indicates the ReLU activation function. Eventually, the feature map with dimension (n,256,256,1) is generated using Ablation-CAM and is resized to (n,32,32) by localising the crucial regions in $S_{\;psp}^{*}$. The resulting resized Ablation-CAM features are depicted as $f_{acam}$.

#### 3.5.4 EfficientNetB7 features

EfficientNetB7 (19) facilitates accurate extraction of hierarchical features through its deep CNN architecture. EfficientNetB7 has seven distinct blocks, of which the Mobile inverted Bottleneck Convolution with a ReLU activation function is considered to be the primary component. Specifically, the compound scaling characteristics of EfficientNetB7 analyse each neural network dimension individually and in a balanced manner to capture high-level spatial and structural features. Thus, the intermediate-level features of the dimension (n,112,112,64) are extracted from the third-layer output of EfficientNetB7 with reduced computational cost and better generalisation. These high-dimensional features are resized to (n,32,32) for effective processing, and accurate atherosclerosis classifications are expressed as $f_{b7}$.

Finally, all these features, such as local frequency descriptor features ($f_{lfd}$), VGG-16 features ($f_{vgg16}$), Ablation-CAM features ($f_{acam}$), and EfficientNetB7 features ($f_{b7}$), are extracted using the pretrained local descriptor and concatenated to generate the feature vector $\left({\bar{F}}_{ext}\right)$ of dimension (n,32,32,4), which is represented in Equation 18 as follows:

$${\bar{F}}_{ext}=f_{lfd}\|\;f_{vgg16}\|\;f_{acam}\|\;f_{b7}$$ (18)

These concatenated best features are subjected to the proposed MemAF-KTCBM model for accurate atherosclerosis classification.

### 3.6 Proposed memory-aware fractional optimisation-enabled deep learning model for atherosclerosis classification

An effective MemAF-KTCBM model is developed for atherosclerosis classification with knowledge distillation characteristics. Even though multiple frameworks are designed for atherosclerosis classification, the existence of certain challenges associated with class imbalance issues (1), data heterogeneity (4), computational complexity (6), and reduced interpretability (5) poses a significant impact on accurate atherosclerosis classification. The MemAF-KTCBM model, thus designed, addresses these downsides by fine-tuning its model parameters using the MemAF optimisation algorithm and a knowledge distillation mechanism.

The proposed MemAF-KTCBM model utilises a knowledge distillation mechanism that incorporates teacher and student models and employs a Kullback–Leibler (KL) divergence loss function. To minimise the difference between the probability distributions of the teacher and student models, the KL divergence loss is employed as an objective function, leading to accurate atherosclerosis classification. The teacher model employs one CNN, two ViTs, and one BiLSTM to train the model for accurate classification. Furthermore, MemAF optimisation is applied to boost classification accuracy and reduce loss. Comparatively, the student model employs a CNN, a BiLSTM, and MemAF optimisation to achieve better atherosclerosis classification with lower loss via the knowledge distillation mechanism. The adoption of the knowledge distillation mechanism in this research effectively extracts the best possible features $\left({\bar{F}}_{ext}\right)$ to acquire crucial disease features. To do so, knowledge distillation resembles a teacher–student model with the Kullback–Leibler divergence loss $\left(X_{kld}\right)$ as its objective function.

**Figure 4:**
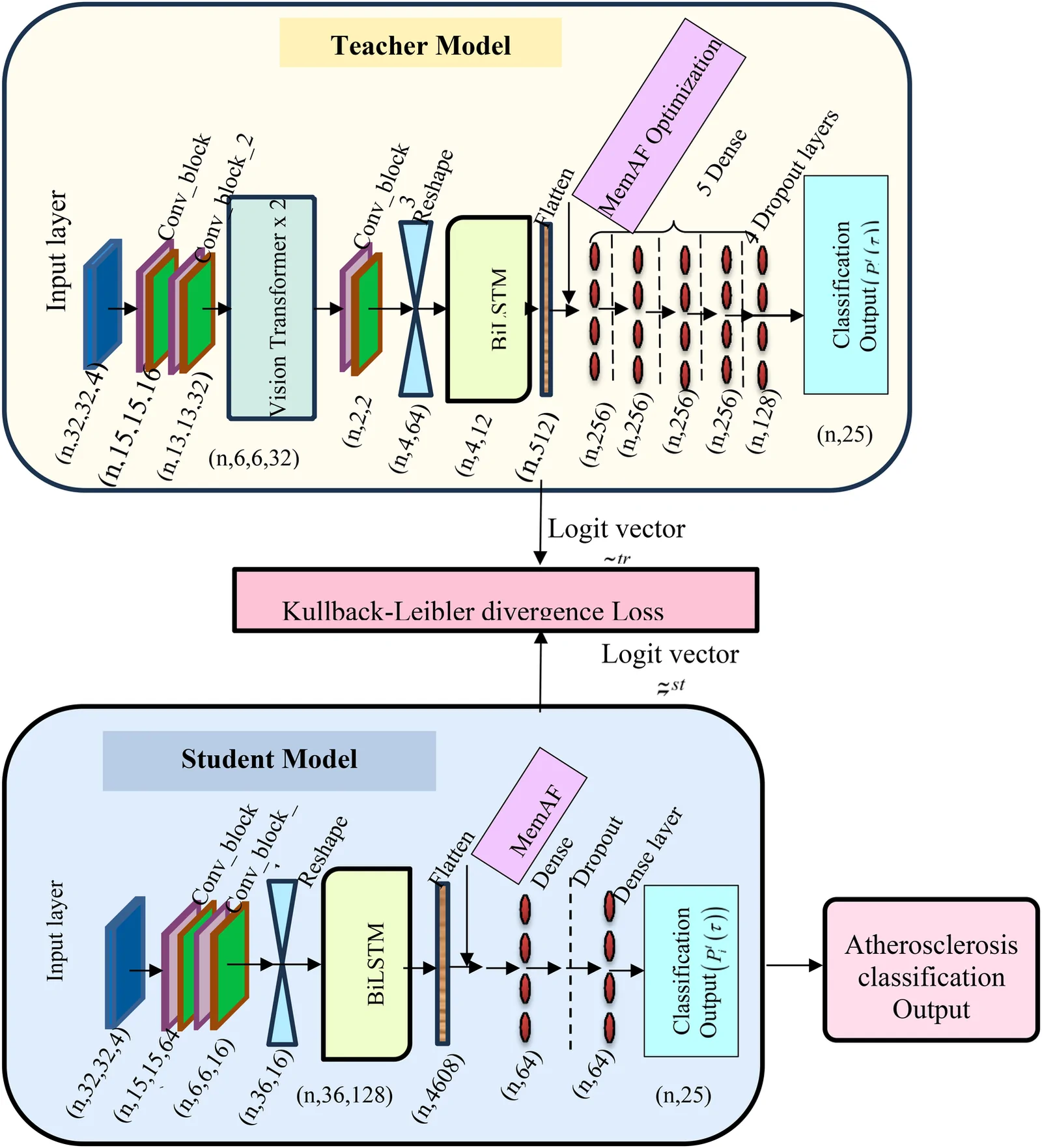
Architecture of the MemAF-KTCBM model.

Herein, the teacher model is much larger than the student model, allowing the latter to mimick the teacher's behavioural features. Fortunately, the smaller student model hardly generates predictions and uncertainties comparable to those of the larger teacher model. The architecture of the MemAF-KTCBM model is shown in Figure 4.

One special type of knowledge distillation is matching logits, where the logit denotes the raw output generated at the last layer of the neural network before the activation function is applied. Accordingly, the logit output of the teacher model is given as ${\tilde{z}}^{tr}$, and that of the student model is given as ${\tilde{z}}^{st}$. Then, the prediction probability of the ith class $\left(P_{i}^{t}(\tau )\right)$ using the softmax activation function with distillation temperature (τ) is given in Equation 19 as follows:

$$P_{i}^{t}(\tau )=\frac{\exp\left(\frac{{\tilde{z}}_{i}^{ttr}}{\tau }\right)}{\sum_{i=1}^{n}\exp\left(\frac{{\tilde{z}}_{i}^{tr}}{\tau }\right)}$$ (19)

where $\sum_{i=1}\exp({\tilde{z}}_{i})$ represents the sum of exponential logits of all classes and ${\tilde{z}}_{i}^{tr}$ indicates the logit of the ith class.

The MemAF-KTCBM is a teacher–student model in which the teacher model consists of an input layer, three convolutional layer blocks, a vision transformer, a reshape layer, a BiLSTM layer, a flatten layer, and five dense layers. The student model comprises an input layer, two convolutional layer blocks, a reshape layer, a BiLSTM layer, a flatten layer, a dropout layer, and two dense layers. To perform accurate atherosclerosis classification, initially, ${\bar{F}}_{ext}$ with dimensions (n,32,32,4) are entered into the input layer, which is provided to the successive convolutional layers. The convolutional layer then extracts crucial informative features $\left({\bar{F}}_{cnv1}\right)$. Through a convolution operation, it aids in the efficient transfer of knowledge to the student model. The formal definition for the convolution operation is given in Equation 20 as follows:

$${\bar{F}}_{cnv1}=℘(\omega *{\bar{F}}_{ext}+\delta )$$ (20)

where ω and δ are the model parameters optimised using the MemAF optimisation algorithm. The crucial informative features then flow from the convolutional layer blocks into the vision transformer (ViT). The ViT (18) identifies key global patterns linked to atherosclerosis by leveraging multiple self-attention mechanisms. Generally, the ViT processes incoming informative features by treating them as sequential tokens. A transformer encoder—comprising several self-attention layers, normalisation layers, and feedforward neural networks, such as a multi-layer perceptron (MLP)—then transforms these tokens into visual embeddings of relevant characteristics by examining their complex relationships. The following Equation 21 shows the functions of the multi-headed attention (MHA) layer.

$$MhA=concat({\bar{h}}_{1},{\bar{h}}_{2},{\bar{h}}_{3},\ldots ,{\bar{h}}_{n})$$ (21)

where ${\bar{h}}_{1},{\bar{h}}_{2},{\bar{h}}_{3},\ldots ,{\bar{h}}_{n}$ denotes the *n* number of heads. Following the MHA layer, the normalisation layer is placed to ensure a stable and constant distribution of activation across the diverse instances in the ViT. This eliminates vanishing-gradient issues and improves generalisation characteristics. The normalised output $\left({\bar{F}}_{norm}\right)$ thus obtained then enters the MLP layer $\left({\bar{F}}_{mlp}\right)$ of the ViT to acquire complex features using the skip connections and two linear transformation layers with the Gaussian error linear unit $\left({℘}_{gelu}\right)$ activation function, is given in Equation 22 as follows:

$${\bar{F}}_{mlp}={℘}_{gelu}(\omega^{T}{\bar{F}}_{norm}+\delta )$$ (22)

Thus, input features of diverse sizes are effectively handled by the ViT, offering better interpretability and scalability. Thus, the intricate features generated by the ViT with dimension (n,6,6,32) are modelled as ${\bar{F}}_{vit}$, which then propagate through the convolutional layer block to generate the feature map of dimension (n,2,2,64). The feature maps are reshaped to (n,4,64) in the successive reshape layer for further processing in the preceding BiLSTM layers. The BiLSTM layer processes the reshaped pertinent features $\left({\bar{F}}_{rs}\right)$ bidirectionally using the input layer, output layer, and forward and backward propagation layers within a single basic unit to generate meaningful features $\left({\bar{F}}_{bilstm}\right)$ of dimension (n,4,128), as given in Equation 23. The forward propagation layer extracts forward features, whereas the backward propagation layer extracts reverse features from front to back (32). Both the forward and reverse features extracted in these layers are integrated in the output layer to generate highly correlated features:

$${\bar{F}}_{bilstm}=[{\vec{\bar{F}}}_{rs}⊕\overset{\leftarrow }{\underset{rs}{\bar{F}}}]$$ (23)

where ${\vec{\bar{F}}}_{rs}$ and $\overset{\leftarrow }{\underset{rs}{\bar{F}}}$ indicate the features obtained after forward and backward processing, respectively. The pertinent features thus obtained with dimension (n,4,128) are flattened to (n,512) in the flattened layer and propagate through five dense blocks comprising dense and dropout layers. The dense layer is provided with a softmax activation function to generate atherosclerosis classification output of dimension (n,25). The dense layer in the teacher model thus aids the better transfer of knowledge from the teacher to the student model, facilitating accurate atherosclerosis classification.

Likewise, ${\bar{F}}_{ext}$ with dimensions (n,32,32,4) are subjected to the input layer of the student model to make accurate atherosclerosis detection and classification based on the knowledge gained from the teacher model. From ${\bar{F}}_{ext}$, the crucial feature maps are generated via by applying a convolution operation in the convolutional blocks. These high-dimensional features are further reshaped in the reshape layer and bidirectionally processed to generate the output features with dimensions (n,32,32,4). The bidirectional features are further flattened in the flatten layer and then processed in the dropout and dense layers with a softmax activation function to provide the classification output, as given in Equation 5. Conceptually, the loss $\left(X_{loss}\right)$ that occurs during the atherosclerosis classification process is analysed via a linear combination of $X_{cce}$ and $X_{kld}$, as given in Equation 7. Thus, the MemAF-KTCBM accurately classifies the diverse classes of atherosclerosis by optimising its parameters via the MemAF optimisation algorithm.

The pseudocode of the proposed MemAF-KTCBM model for atherosclerosis classification is detailed in Algorithm 1.

Algorithm 1 **Pseudocode of the proposed MemAF-KTCBM model.**

Input: Patient medical Image ($X_{i}$)

Output: Classified Atherosclerosis disease

Begin preprocessing ($X_{i}$)

   enhanced image=gamma correction ($X_{i}$)

   return enhanced image ($X_{i}^{*}$)

augmentation ($X_{i}^{*}$)

   MemAF-SGAN= {generator (), discriminator ()}

   Output= MemAF-SGAN ($X_{i}^{*}$)

   return augmented image ($S_{gan}$)

   Resized image=resize_image ($S_{gan}$)

   Greyscale image = greyscale conversion ($S_{gan}$)

   return grey-scale image ($S_{gry}^{*}$)

segmentation ($S_{gry}^{*}$)

         MPSPNet= {

              Conv_block ()

              Conv_block ()

              MemAF optimization ()

              Pyramid pooling ()

              Conv2d ()

                        }

        Segmented output= MPSPNet $\left(S_{psp}^{*}\right)$

feature extraction $\left(S_{psp}^{*}\right)$

          $f_{lfd}$= Local Frequency Descriptor $\left(S_{psp}^{*}\right)$

         $f_{vgg16}$= VGG-16$\left(S_{psp}^{*}\right)$

           $f_{acam}$= Ablation-CAM $\left(S_{psp}^{*}\right)$

            $f_{b7}$ = EfficientNetB7$\left(S_{psp}^{*}\right)$

             Final features$\left({\bar{F}}_{ext}\right)$=$f_{lfd}\|f_{vgg16}\|f_{acam}\|f_{lfd}\|f_{b7}$

              return concatenated features$\left({\bar{F}}_{ext}\right)$

Procedure Proposed model (Train data, Test data)

            MemAF-KTCBM model= {Teacher model {

                              Conv ()

                              Vision Transformer ()

                              Conv ()

                              MemAF optimization ()

                              Dense ()

                              Output ()

                              }

                       Student model {

                              Conv ()

                              Conv ()

                              MemAF optimization ()

                              Dense ()

                              Output ()

                              }

                        }

        Trained model = Knowledge distillation (MemAF-KTCBM)

Procedure: Testing model = trained model (Test data)

        return output

def Xilinx (MemAF-KTCBM)

        Xmodel=VAI (MemAF-KTCBM)-Vitis AI

        return model.

#### 3.6.1 Memory-aware fractional optimisation algorithm

The MemAF optimisation algorithm is designed for the MemAF-KTCBM-based atherosclerosis classification model, which mimics the exploration characteristics of the low-frequency sensing nature of the Sand Cat Swarm Optimisation (24) and the random exploitation characteristics of the Serval Optimisation Algorithm (23) to avoid getting trapped in local optima. Specifically, the strong hearing, sensing, and jumping characteristics of the Sand Cat in attacking the prey are hybridised within the Serval Cat algorithm for accurate atherosclerosis classification. Despite the availability of various optimisation approaches, the persistence of certain limitations, including stagnation issues, premature convergence, and local optima, has limited their widespread use in the optimisation process. Hence, the MemAF optimisation algorithm uses fractional calculus theory to eliminate such discrepancies and attain optimal atherosclerosis classification performance. For image augmentation, the MemAF optimisation algorithm is applied before the last convolutional layer of the MemAF-SGAN, whereas it is applied before the pyramid pooling module in the MPSPNet. For accurate atherosclerosis classification with the MemAF-KTCBM, the MemAF optimisation algorithm is applied after the convolutional layer of the MemAF-KTCBM. The mathematical expression of the MemAF optimisation algorithm is detailed further.

***Initialisation:*** To begin the MemAF optimisation algorithm, all randomly generated solutions $\left(W_{i}\in \omega ,\delta \right)$ in the search space are initialised, as given in Equation 24:

$$W_{i}:W_{i,j}=c_{j}+rnd\cdot (d_{j}-c_{j});\;i=1,2,3,\ldots ,K;\;j=1,2,3,\ldots ,D$$ (24)

where *K* refers to the maximum number of solutions, *D* denotes the dimension of the search space, $W_{i}$ denotes the initial position vector of the ith solution, rnd indicates the random value ranging between [0,1], and $c_{j}$ and $d_{j}$ are the lower and upper bounds of the search space with jth dimension, respectively.

***Fitness evaluation:*** After initialising all solutions in the search space, the fitness value of each solution is determined to ensure the highest-performing solutions in the optimisation process. For MemAF-SGAN, fitness evaluation $\left(\tilde{F}(W_{i})\right)$ takes place using a multi-objective function that combines the maximum of accuracy $\left(A_{cc}\right)$, sensitivity $\left(S_{en}\right)$, and specificity $\left(S_{\;pec}\right)$, as modelled in the following Equation 25:

$$\tilde{F}(W_{i})=\max\left(\frac{A_{cc}+S_{en}+S_{\;pec}}{3}(W_{i})\right)$$ (25)

For MPSPNet-based segmentation, fitness evaluation is based on a combination of the Dice similarity coefficient $\left(D_{ice}\right)$ and the Jaccard index $\left(J_{ac}\right)$, as given in the following Equation 26:

$$\tilde{F}(W_{i})=\max\left(\frac{D_{ice}+J_{ac}}{2}(W_{i})\right)$$ (26)

while the MemAF-SGAN-based image augmentation evaluates fitness value using the maximum accuracy, as given in the following Equation 27:

$$\tilde{F}(W_{i})=\max(A_{cc}(W_{i}))$$ (27)

***Parameter assumption:*** Upon successful evaluation of the fitness function, a crucial parameter, the transition control parameter R, is introduced to provide better control over the exploration and exploitation characteristics of the solutions in the search space. The transition control parameter R is formally defined in Equation 28:

$$R=2\times J_{guide}\times rnd(0,1)-J_{guide}$$ (28)

where $J_{guide}$ represents the the guiding parameter which is given in Equation 29:

$$J_{guide}=J_{\;fb}-\left(\frac{2\times J_{\;fb}\times t}{t_{\max}+t_{\max}}\right)$$ (29)

where $J_{\;fb}$ denotes the feedback factor and *t* and $t_{\max}$ indicate the current and maximum iterations, respectively. In addition, the range factor $\left(\bar{r}\right)$ unveils the sensitivity range of each solution in both the exploration and exploitation phases. Hence, the formula for computing the sensitivity range using $\bar{r}$ is given in Equation 30 as follows:

$$\bar{r}=J_{guide}\times rnd(0,1)$$ (30)

Fortunately, the optimal solution-search strategy for fine-tuning the model parameters falls into two distinct cases: exploration via the historical updating phase and the angle-modulation-based exploitation phase. The two distinct cases are detailed further.

***Case (i)—Exploration* via *the historical updating phase*** |R|>1*:* The solution makes use of the historical updating phase to enter the exploration stage. In this stage, the transition control parameter is greater than unity. Hence, a detailed scan is performed to obtain the optimal solution by enhancing its global search characteristics. Hence, the annotation for the updated position is given in Equation 31 as follows:

$$W_{i}^{t+1}=W_{i}^{t}+\bar{r}(W_{best}^{t}-(rnd(0,1)+\alpha_{2})W_{i}^{t})$$ (31)

where $\alpha_{2}$ represents the random number selected from the set {1,2}. The detailed scan is provided to make a global search. However, the characteristics cause the solution to move blindly, without considering its own performance itself or its neighbours, which affects overall performance. Hence, the MemAF optimisation algorithm incorporates the fractional calculus theory to find an optimal solution, expressed as follows:

$$W_{i}^{t+1}-W_{i}^{t}=\bar{r}(W_{best}^{t}-(rnd(0,1)+\alpha_{2})W_{i}^{t})$$ (32)

By applying fractional calculus to the left-hand side of Equation 32, the solution update rule can be reformulated, as shown in Equation 33:

$$\delta^{\alpha_{3}}(W_{i}^{t})=\bar{r}(W_{best}^{t}-(rnd(0,1)+\alpha_{2})W_{i}^{t})$$ (33)

The representation of the updated solution using the fractional calculus theory is mathematically given in Equation 34:

$$\begin{array}{rl} & W_{i}^{t+1}=\alpha_{3}W_{i}^{t}+\frac{1}{2}\alpha_{3}W_{i}^{t-1}+\frac{1}{6}\alpha_{3}(1-\alpha_{3})W_{i}^{t-2}+\frac{1}{24}\alpha_{3}(1-\alpha_{3})(2-\alpha_{3})W_{i}^{t-3}\\ & \;\;\;+\bar{r}(W_{best}^{t}-(rnd(0,1)+\alpha_{2})W_{i}^{t}) \end{array}$$ (34)

where $\alpha_{3}$ denotes the random factor ranging between [0,1]. The memory-driven search-dynamics nature of fractional calculus theory forces the solution to learn more from historical behaviour, which further reduces erratic jumps, improves stability, and eliminates chaotic oscillations by attaining global optima.

***Case (ii)***—***Angle-modulation-based exploitation phase*** |R|<1: The solution enters the angle-modulation-based exploitation phase when the transition control parameter is less than unity. Thereby, the position of the solution is updated as shown in Equation 35:

$$W_{i}^{t+1}=W_{gbl}-\bar{r}(W_{rnd}\cdot \cos(\theta ))$$ (35)

where $W_{rnd}$ represents the random position of the solution, $W_{gbl}$ indicates the global search position, and cos⁡(θ) is the random cosine angle, which is formulated as follows in Equations 36 and 37:

$$W_{gbl}=W_{best}^{t}+\frac{\alpha_{4}(d_{j}-c_{j})}{t}$$ (36)

$$W_{rnd}=|rnd(0,1)\cdot W_{best}^{t}-W_{i}^{t}|$$ (37)

where $W_{gbl}$ represents the global search position, $W_{best}^{t}$ denotes the best candidate solution obtained at the tth iteration, $W_{rnd}$ refers to the randomly generated search position, $W_{i}^{t}$ indicates the current position of the tth solution, $d_{j}$ and $c_{j}$ denote the upper and lower boundary limits of the jth search dimension, respectively, $\alpha_{4}$ represents a random control coefficient, and rnd(0,1) generates a uniformly distributed random value in the range (0,1).

**Figure 5:**
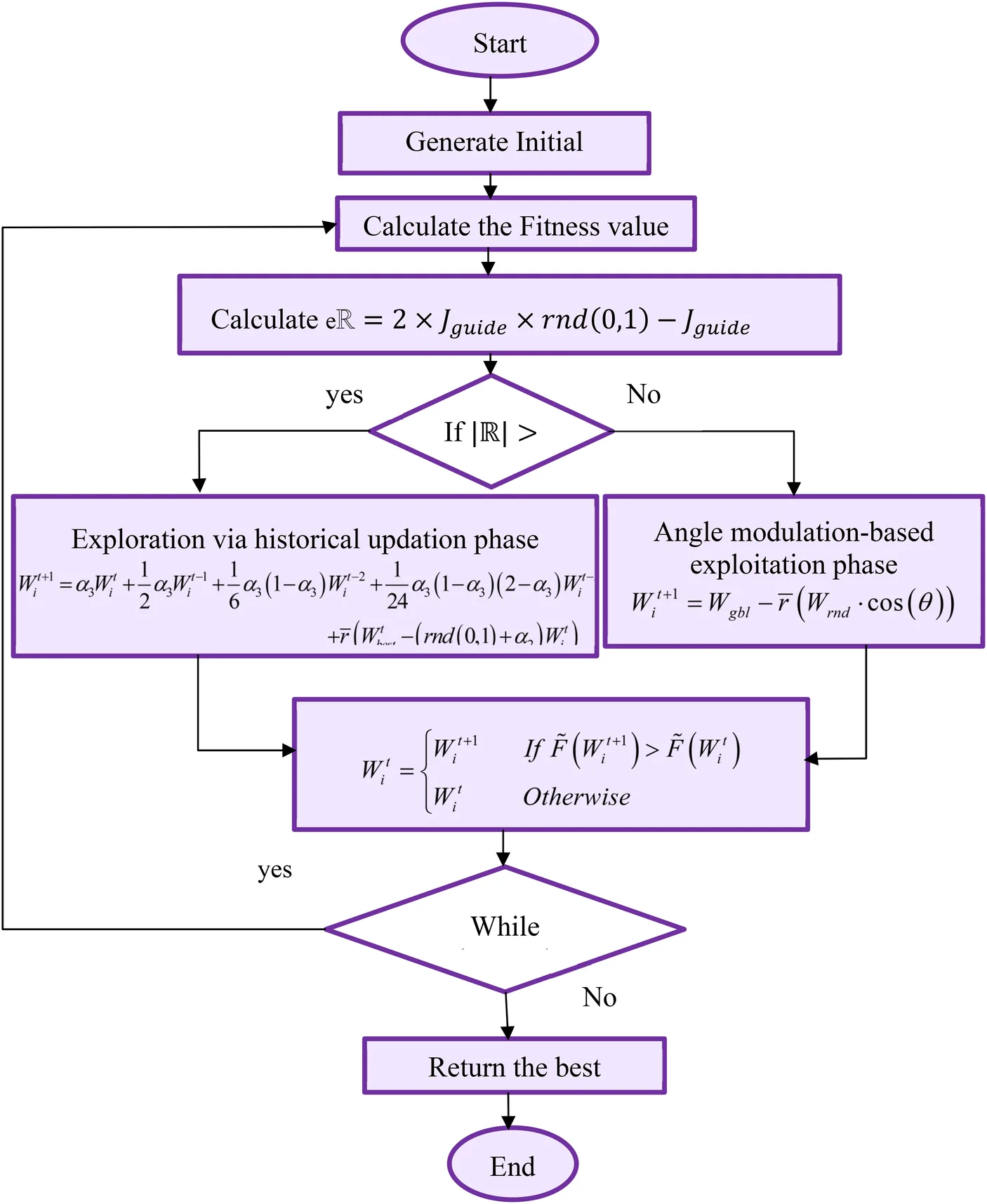
Flowchart representation of MemAF optimisation.

***Termination:*** The above process is repeated until the termination condition $\left(t<t_{\max}\right)$ is satisfied. The MemAF approach returns the current solution as the globally optimal solution once the termination condition is satisfied. The flowchart representation for MemAF optimisation is shown in Figure 5.

## 4 Results and discussion

The atherosclerosis classification performance of the MemAF-KTCBM is assessed in this section by varying the training percentage from 40% to 90% and the *K*-fold value from 6 to 10. The evaluation of the results is detailed as follows.

### 4.1 Experimental setup

The MemAF-KTCBM model is implemented in Python 3.8.9 and Xilinx to perform accurate atherosclerosis classification on Windows 11. Here, a 16 GB RAM is used for effective classification at a 1.7 GHz clock frequency. The following are the steps involved in the research experiment.

- Freeze the saved model using the TensorFlow tools to generate the TensorFlow frozen graph (.pb),
- Prepare a calibration dataset for INT8 quantisation,
- Run the Vitis AI quantiser on the frozen graph or saved model to generate the quantised model,
- Verify the quantised model and the target DPU architecture file (arch.json), and
- Obtain the final .xmodel file as output from the compiler, which is ready to be deployed on the DPU.

The initial parameters of the MemAF-KTCBM model include a learning rate of 0.001, a batch size of 32, the number of hidden units of 100, a dropout rate of 0.5, activation function “ReLU”, the number of convolutional layers of 4, the number of neurons in the layer of 5, the number of filters of (32, 64, 238, 356, 512), loss function Kullback–Leibler divergence loss, and regularisation with a value of 0.001, as well as distributed architecture and the default optimiser Adam. Moreover, the input data are split into several training–test ratios, such as 40:60, 50:50, 60:40, 70:30, 80:20, and 90:10. The MemAF-KTCBM model achieved its peak performance at a 90:10 ratio, ensuring that patient-level separation is maintained across training and test datasets.

### 4.2 Dataset description

For accurate atherosclerosis classification, the MemAF-KTCBM uses two distinct datasets: ARCADE (29) and CADICA (30). All input x-ray angiographic images were resized to 512 × 512 × 3 before training.

**ARCADE dataset** (29)**:** The ARCADE (automatic region-based coronary artery disease diagnostics using x-ray angiographic images) dataset has two folders, each containing 300 x-ray angiographic images stored in COCO format, totalling 451.7 MB. Syntax and Stenosis are the top-level directories of this dataset, encompassing various files for vessel branch classification and stenosis detection. This dataset contains 25 classes: 1, 2, 3, 4, 5, 6, 7, 8, 9, 9a, 10, 10a, 11, 12, 12a, 13, 14, 14a, 15, 16, 16a, 16b, 16c, 12b, 14b, which are expressed in Equation 5; a total of 7,500 samples are used for the atherosclerosis classification. The dataset was previously annotated by medical experts and contributes to predicting automatic risk assessments for coronary artery disease. The input dataset was first divided into 80% training data and 20% test data before applying the MemAF-SGAN-based augmentation. Only the training data were used for GAN training and synthetic image generation, while the test data remained unchanged and were used exclusively for model evaluation, thereby preventing data leakage.

**CADICA dataset** (30)**:** The coronary artery disease dataset is manually annotated from invasive coronary angiographic images of 42 patients and was acquired from the Hospital Universitario Virgen de la Victoria, Spain. The dataset comprises videos of the patients and related disease metadata stored in PNG files, with a total storage of 2.86 GB. This dataset contains three classes: non-ST-segment elevation acute coronary syndrome, ST-segment elevation acute coronary syndrome, and stable angina, with a total of 1,376 samples used for the proposed atherosclerosis classification research. Moreover, the research used a total of 15,000 images for atherosclerosis classification.

**Figure 6:**
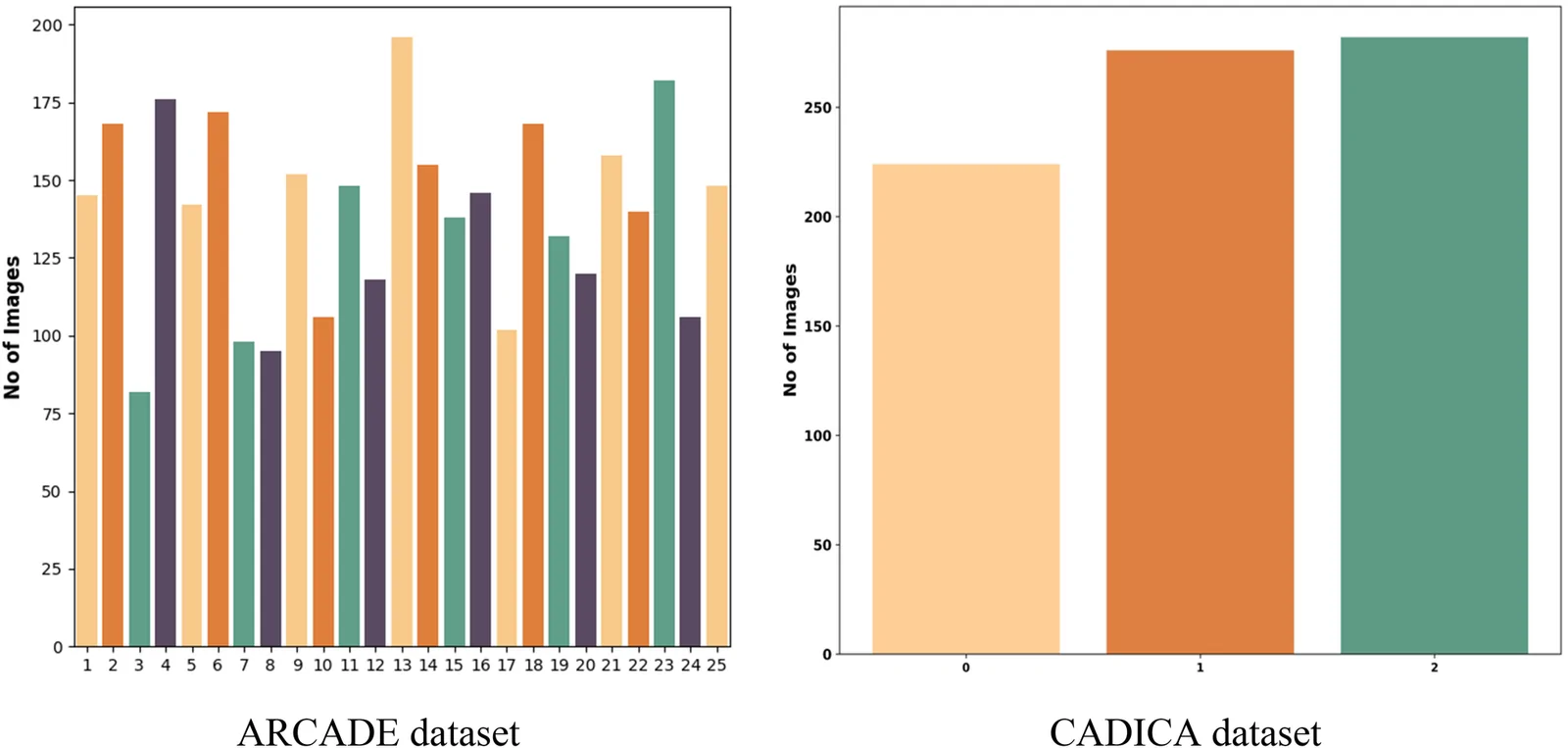
Dataset visualisation.

Figure 6 illustrates the classwise distribution of the datasets used for atherosclerosis classification. In the ARCADE dataset (left), the light orange, orange, green, and purple bars are used to visually distinguish the distribution of angiographic images across the 25 coronary artery branch classes (1, 2, 3, 4, 5, 6, 7, 8, 9, 9a, 10, 10a, 11, 12, 12a, 12b, 13, 14, 14a, 14b, 15, 16, 16a, 16b, and 16c). The colours are used solely for visual differentiation of the class frequencies and do not indicate any additional clinical information. In the CADICA dataset (right), the light orange, orange, and green bars correspond to the three diagnostic categories, namely, non-ST-segment elevation acute coronary syndrome (NSTE-ACS), indicated by 0; ST-segment elevation acute coronary syndrome (STE-ACS), indicated by 1; and stable angina, indicated by 2, illustrating the number of angiographic images available in each class.

### 4.3 Performance metrics

Accuracy, negative predictive value (NPV), recall, Matthews correlation coefficient (MCC), positive predictive value (PPV), and precision are the diverse metrics used to assess the atherosclerosis classification performance of the MemAF-KTCBM. Each metric is detailed with its formula in Table 2, where $R_{tp}$ represents the true positives, $R_{tn}$ indicates the true negatives, $R_{\;fp}$ denotes the false positives, and $R_{\;fn}$ represents the false negatives.

**Table 2:** Details of performance metrics.

Metrics	Formula	Description
Accuracy $\left(X_{acc}\right)$	$X_{acc}=\frac{R_{tp}+R_{tn}}{R_{tp}+R_{tn}+R_{\;fp}+R_{\;fn}}$	The model's ability to correctly detect and classify atherosclerosis
Precision $\left(X_{\;prec}\right)$	$X_{\;prec}=\frac{R_{tp}}{R_{tp}+R_{\;fp}}$	An accurate measure of the positive predictions
Recall $\left(X_{rec}\right)$	$X_{rec}=\frac{R_{tp}}{R_{tp}+R_{\;fn}}$	An accurate measure of the actual true positives
NPV $\left(X_{npv}\right)$	$X_{npv}=\frac{R_{tn}}{R_{tn}+R_{\;fn}}$	The probability measure of the actual true negatives
PPV $\left(X_{\;ppv}\right)$	$X_{\;ppv}=\frac{R_{tp}}{R_{tp}+R_{\;fp}}$	The probability measure of the actual true positives
MCC $\left(X_{\;ppv}\right)$	$X_{mcc}=\frac{\left(R_{tp}\cdot R_{tn})-(R_{\;fp}\cdot R_{\;fn}\right)}{\sqrt{\left(R_{tp}+R_{\;fp})(R_{tp}+R_{\;fn})(R_{tn}+R_{\;fp})(R_{tn}+R_{\;fn}\right)}}$	Robust binary classification performance, especially in imbalanced datasets, is achieved by analysing the correlation between the actual and predicted outputs

### 4.4 Experimental results

**Figure 7:**
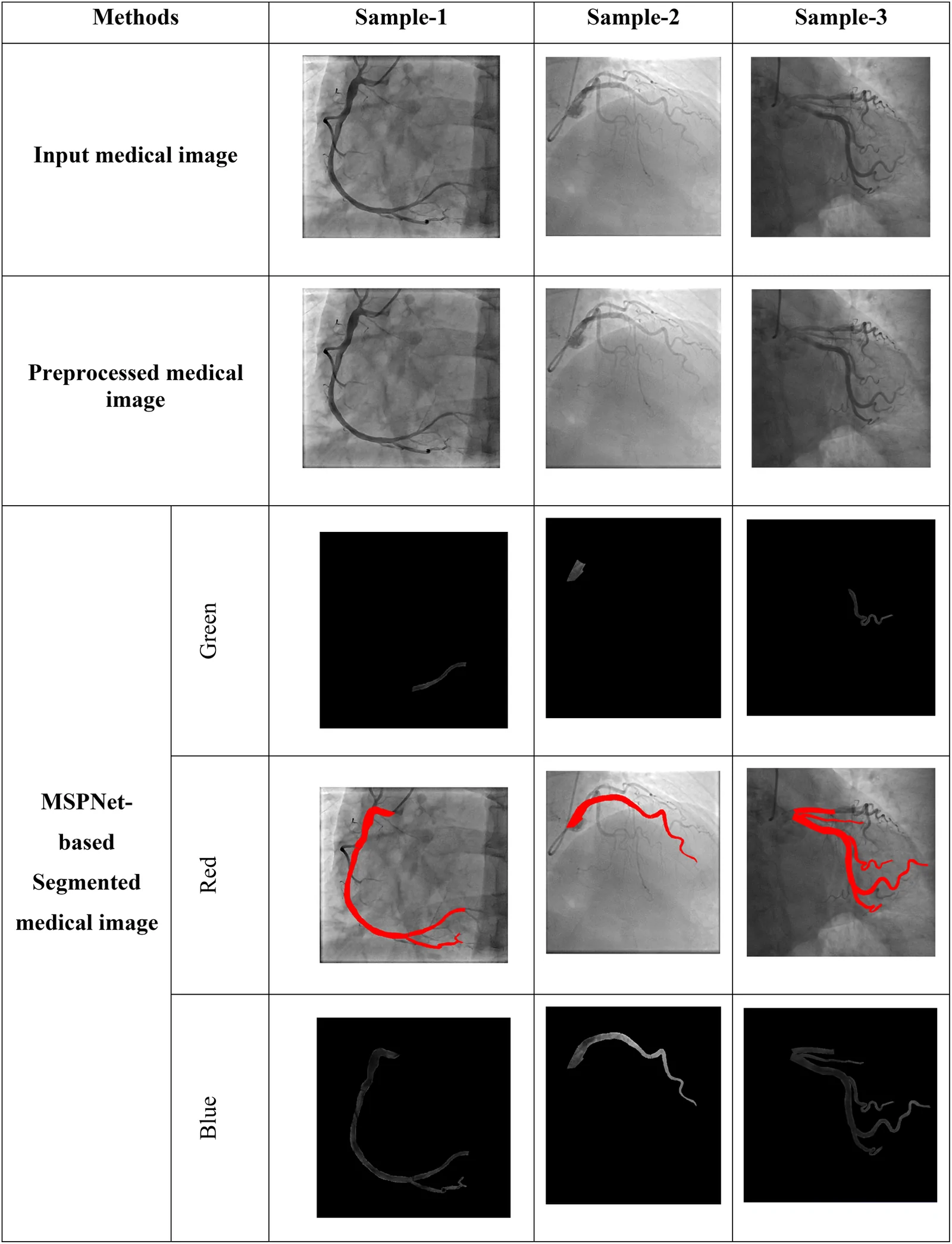
Experimental results of MemAF-KTCBM.

The experimental results of the MemAF-KTCBM for atherosclerosis classification are presented in Figure 7. Figure 7 shows the input medical image, the preprocessed output, the MSPNet-based segmented medical image, the extracted diverse features, and the atherosclerosis classification results.

**Figure:**
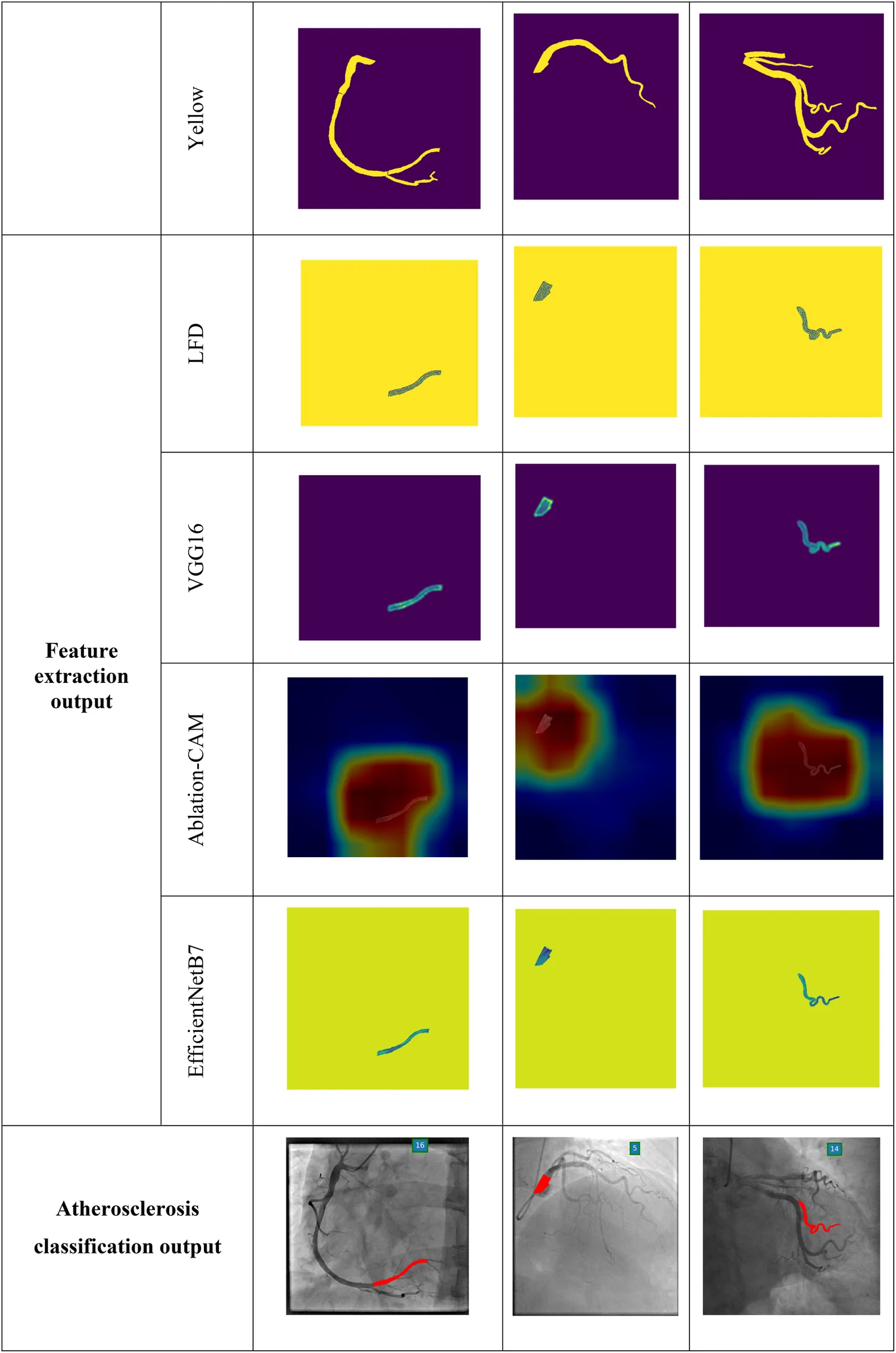


### 4.5 Performance analysis

The atherosclerosis classification performance of the MemAF-KTCBM is briefly explained in this subsection by varying the training percentage from 40, 50, 60, 70, 80, and 90 at diverse epoch sizes for the ARCADE and CADICA datasets.

Tables 3, 4 exemplify the atherosclerosis classification performance of the MemAF-KTCBM using the ARCADE and CADICA datasets for various training percentages and *K*-fold values. At a 90% training split, the MemAF-KTCBM detects and classifies atherosclerosis with 97.73% accuracy, 98.14% precision, 0.98 NPV, 96.91% recall, 0.98 PPV, and 0.95 MCC with 500 epochs. From the analysis, it is concluded that the best possible feature extraction using the pretrained local descriptor accelerates the atherosclerosis classification process, improves interpretability, and reduces model complexity.

**Table 3:** Performance analysis of the model based on the ARCADE dataset at 90% training percentage for multiple epochs.

At 90% training	Accuracy (%)	MCC	NPV	PPV	Precision (%)	Recall (%)
MemAF-KTCBM at epoch = 100	90.17	0.88	0.90	0.90	89.89	90.75
MemAF-KTCBM at epoch = 200	92.87	0.91	0.92	0.92	92.12	94.38
MemAF-KTCBM at epoch = 300	93.20	0.94	0.93	0.93	92.57	94.46
MemAF-KTCBM at epoch = 400	94.81	0.94	0.95	0.95	94.72	94.97
MemAF-KTCBM at epoch = 500	98.64	0.96	0.99	0.99	99.06	97.82

**Table 4:** Performance analysis using the CADICA dataset at 90% training percentage for multiple epochs.

At 90% training	Accuracy (%)	MCC	NPV	PPV	Precision (%)	Recall (%)
MemAF-KTCBM at epoch = 100	89.26	0.87	0.89	0.89	88.98	89.83
MemAF-KTCBM at epoch = 200	92.00	0.90	0.91	0.91	91.25	93.50
MemAF-KTCBM at epoch = 300	92.33	0.93	0.92	0.92	91.71	93.58
MemAF-KTCBM at epoch = 400	93.93	0.93	0.94	0.94	93.85	94.09
MemAF-KTCBM at epoch = 500	97.73	0.95	0.98	0.98	98.14	96.91

### 4.6 Comparative analysis

Here, the atherosclerosis classification performance of the MemAF-KTCBM is evaluated by analysing its superiority over the traditional UNet (25), redundancy feature diminishment strategy (RFDS) (26), three-dimensional CNN (3D-CNN) (27), dense network (DenseNet)-201 (28), Sand Cat Swarm Optimisation-based KMTCN (SCSO) (24), and Serval Optimisation Algorithm (SOA)-KMTCN (23). To provide a fair comparison, the comparative methods are retrained with the proposed MemAF-KTCBM model and evaluated under identical parameter settings, with training percentages ranging from 40% to 90% and *K*-fold values ranging from 6 to 10, as described in the following.

#### 4.6.1 Comparative analysis using the ARCADE dataset based on training percentage

**Figure 8:**
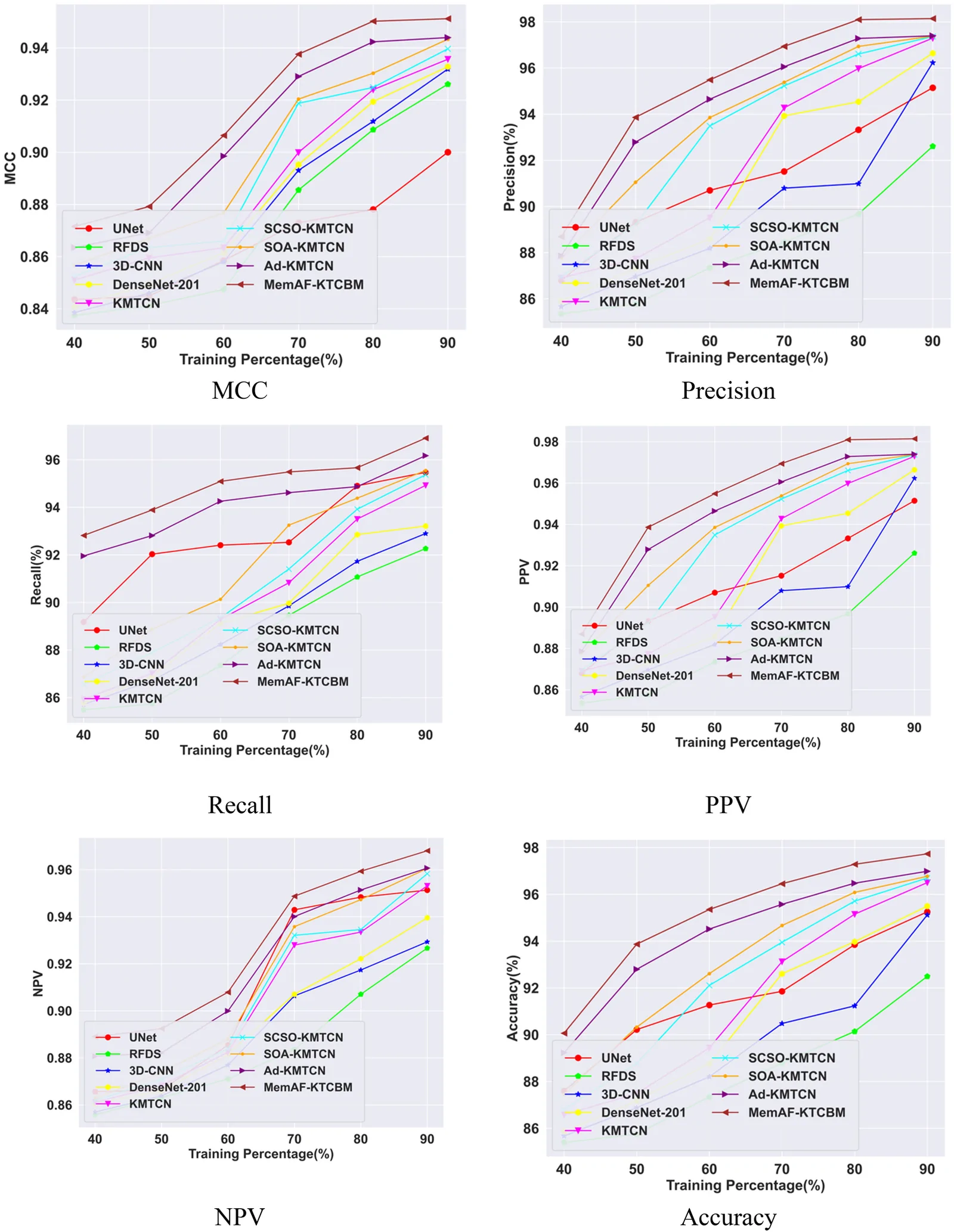
Comparative analysis based on training percentage using the ARCADE dataset.

Figure 8 shows a comparative evaluation of the MemAF-KTCBM model against traditional methods for atherosclerosis classification using the ARCADE dataset across varying training percentages. With 98.64% accuracy at a 90% training split, the MemAF-KTCBM outperforms the conventional UNet, RFDS, and 3D-CNN by 2.16%, 5.02%, and 2.35%, respectively. In terms of precision, the MemAF-KTCBM achieves 99.05%, surpassing the existing SCSO-KMTCN by 0.58% and SOA-KMTCN by 0.57%. In terms of the recall metric, the MemAF-KTCBM achieves 97.81%, representing a performance difference of 14.43% against conventional RFDS, 3.82% against 3D-CNN, and 3.55% against DenseNet-201. The MemAF-KTCBM achieves 0.97 NPV, 0.99 PPV, and 0.96 MCC. Hence, the comparative analysis assesses the impact of segmenting only cardiac structures using MPSPNet in the MemAF-KTCBM-based atherosclerosis classification process, leveraging its multi-scale contextual information aggregation characteristics.

#### 4.6.2 Comparative analysis using the ARCADE dataset based on *K*-fold

**Figure 9:**
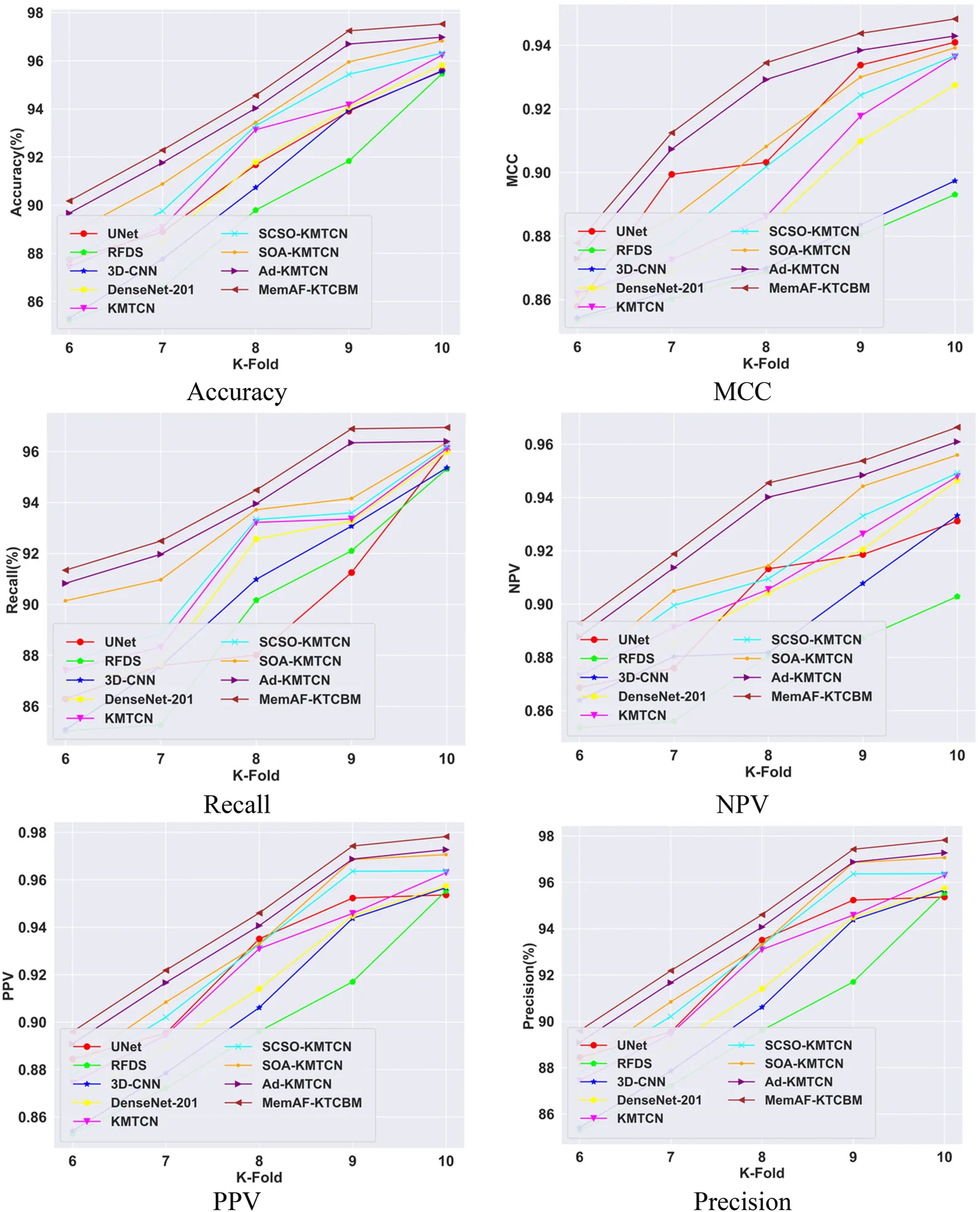
Comparative analysis using *K*-fold cross-validation on the ARCADE dataset.

Figure 9 shows the atherosclerosis classification performance of the MemAF-KTCBM using the ARCADE dataset for various *K*-fold values. At 10-fold cross-validation, the MemAF-KTCBM attains an accuracy of 97.53%, surpassing the traditional UNet by 1.98%, RFDS by 2.11%, and 3D-CNN by 2.01%. Furthermore, the MemAF-KTCBM achieves 97.82% precision and 96.94% recall, surpassing traditional UNet, which achieves 2.51% precision and 0.91% recall. With an NPV of 0.96, the MemAF-KTCBM outperforms the conventional UNet and RFDS by 3.64 NPV and 6.57 NPV, respectively. Moreover, the 0.98 PPV and 0.95 MCC attained using the MemAF-KTCBM in atherosclerosis classification at 10-fold cross-validation are also higher than those of conventional RFDS by 2.34 PPV and 5.82 MCC and of 3D-CNN by 2.20 PPV and 5.37 MCC. This evaluation makes it clear that effective tuning of the MemAF-KTCBM parameters using the MemAF approach improves its classification performance by eliminating premature convergence issues. Furthermore, with an NPV of 0.96, the MemAF-KTCBM outperforms conventional 3D-CNN by 3.99 and DenseNet-201 by 2.94. In addition, the 0.95 PPV and 0.95 MCC of the MemAF-KTCBM are higher than those of the existing UNet by 3.05 and 5.38, respectively. Hence, the intensely deeper architecture of the MemAF-KTCBM, along with a knowledge distillation mechanism, aids in the accurate identification of complex disease patterns.

#### 4.6.3 Comparative analysis using the CADICA dataset based on training percentage

**Figure 10:**
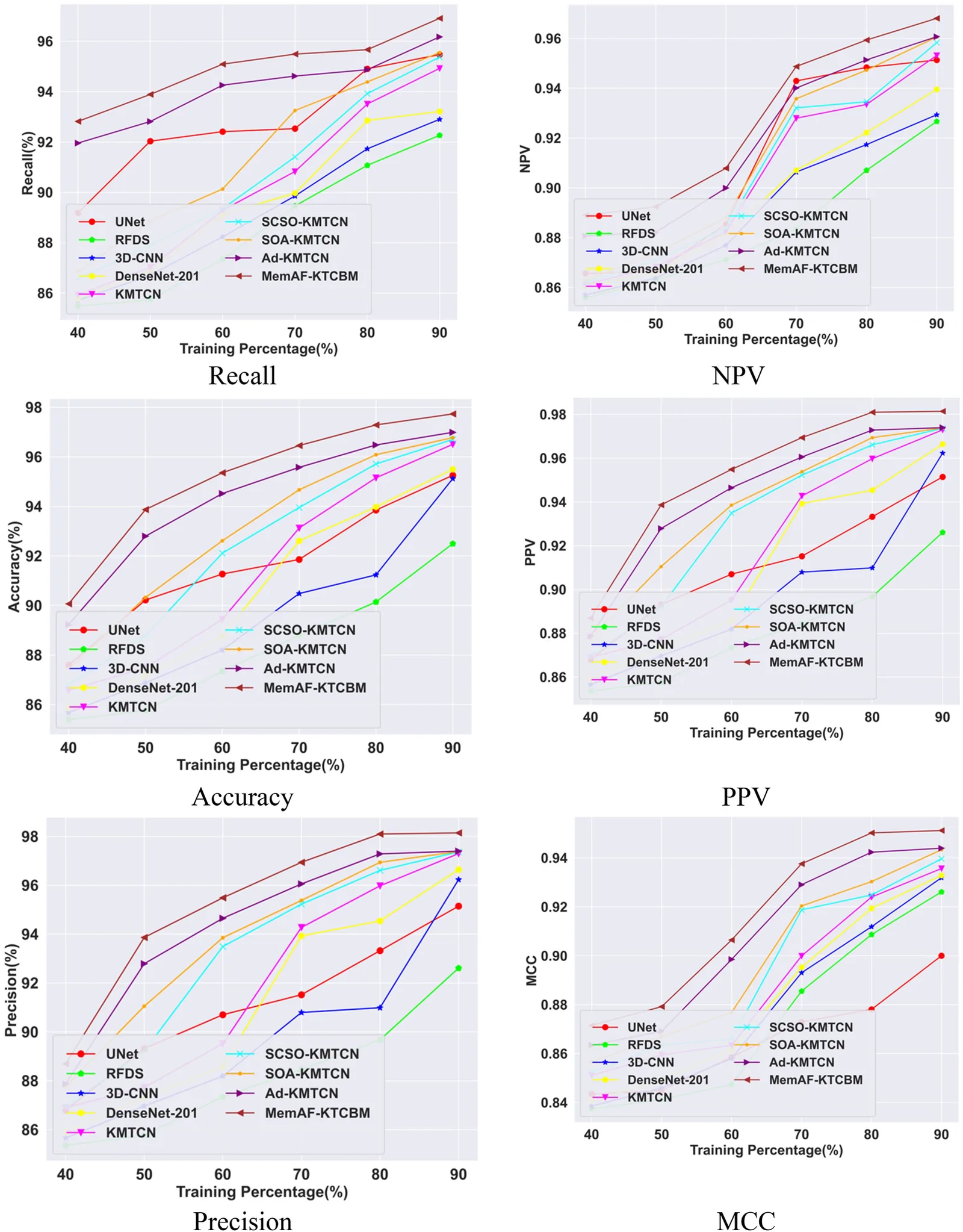
Comparative analysis based on training percentages using the CADICA dataset.

The comparative performance of the MemAF-KTCBM model across varying the training percentages using the CADICA dataset is illustrated in Figure 10. Here, the MemAF-KTCBM achieves 97.73% accuracy at a 90% training split, which surpasses the conventional UNet and RFDS by 2.53% and 5.35%, respectively. In terms of recall, the MemAF-KTCBM reaches 96.91%, exceeding the recall of traditional 3D-CNN by 4.13% and DenseNet-201 by 3.82%. Achieving a precision of 98.14%, the MemAF-KTCBM outperforms existing UNet by 3.05% and RFDS by 5.64%. Hence, the MemAF-KTCBM model, along with a knowledge distillation mechanism, aids in the accurate identification of complex disease patterns. Furthermore, in terms of NPV, PPV, and MCC, MemAF-KTCBM achieves scores of 0.97, 0.98, and 0.95, respectively, surpassing other existing models. Hence, the MemAF-SGAN model demonstrates superior performance in atherosclerosis classification.

#### 4.6.4 Comparative analysis using the CADICA dataset based on *K*-fold

**Figure 11:**
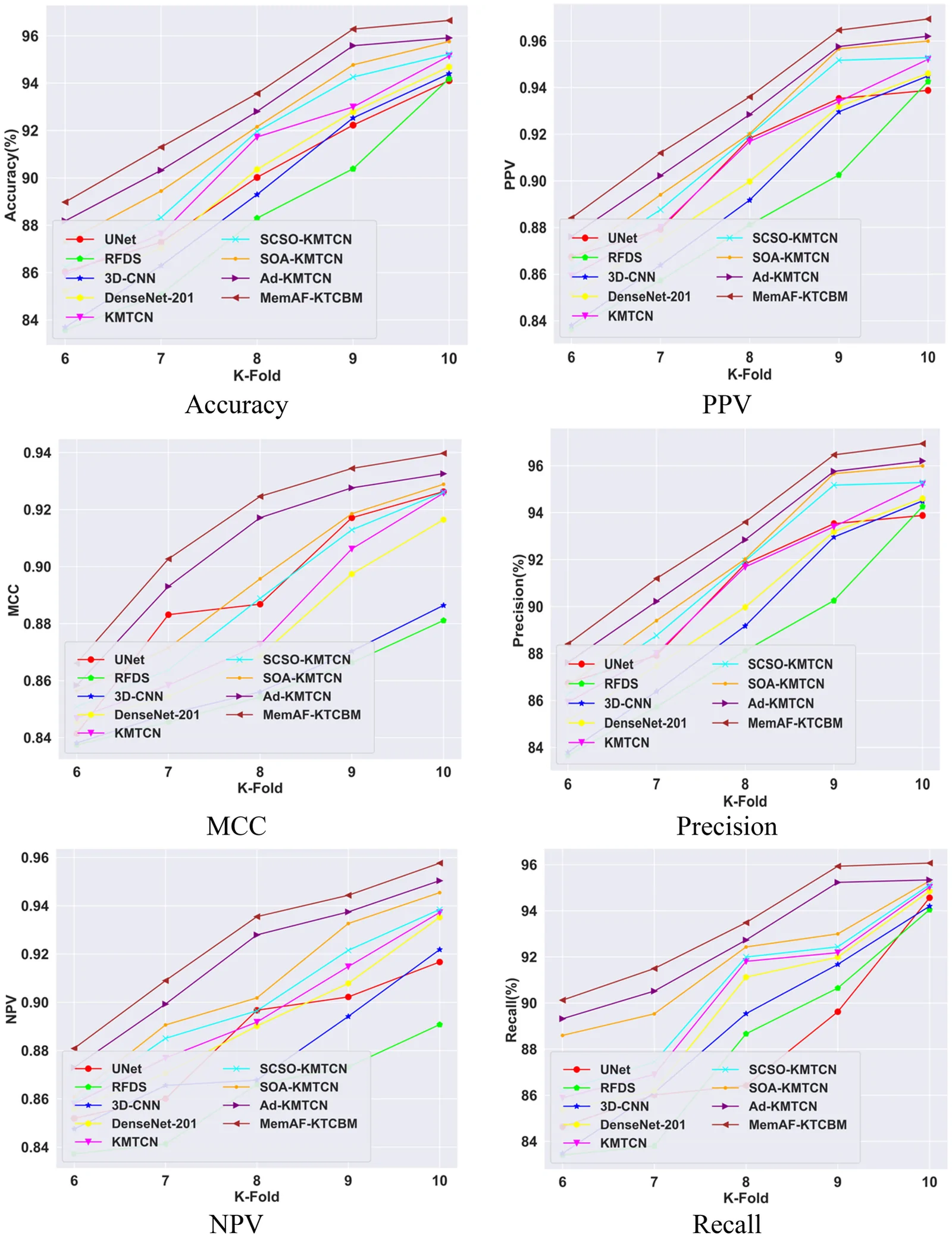
Comparative analysis using *K*-fold cross-validation on the CADICA dataset.

Figure 11 shows the *K*-fold analysis of the MemAF-KTCBM model compared with the traditional atherosclerosis classification frameworks. Here, the MemAF-KTCBM achieves 96.64% accuracy in atherosclerosis classification, surpassing existing UNet by 2.63%, RFDS by 2.55%, and 3D-CNN by 2.33%. Furthermore, the MemAF-KTCBM achieves 96.94% precision, exceeding traditional 3D-CNN, DenseNet-201, and KMTCN by 2.52%, 2.41%, and 1.78%, respectively. In terms of recall, the MemAF-KTCBM exceeds the standard SCSO-KMTCN by 0.98%, SOA-KMYCN by 0.80%, and DenseNet-201 by 1.27%, with 96.07% recall. In terms of NPV, PPV, and MCC, the MemAF-KTCBM achieves scores of 0.96, 0.97, and 0.94, respectively. The metric values thus obtained surpass the RFDS by 6.98 in NPV, 2.77 in PPV, and 6.24 in MCC. Thus, it is apparent that using quality-improved medical images for atherosclerosis classification improved classification performance by generating diverse data with MemAF-SGAN, yielding better generalisability.

### 4.7 Convergence analysis

**Figure 12:**
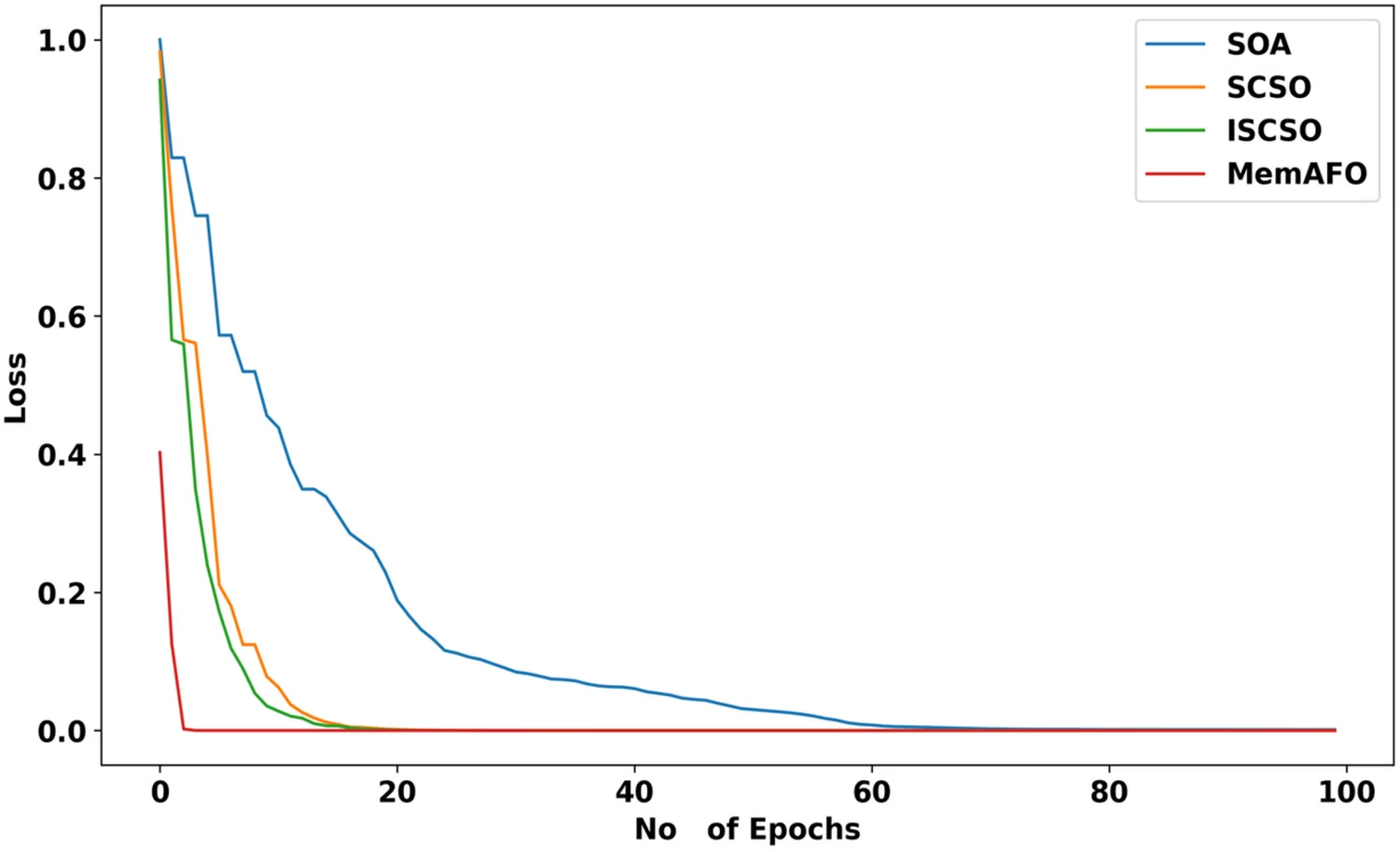
Comparison of convergence analysis of the MemAF optimisation algorithm with those of other existing algorithms.

The convergence analysis of the MemAF optimisation algorithm is compared with other existing algorithms and visualised in Figure 12. Traditional optimisation algorithms often suffer from high computational overhead, poor convergence, and getting trapped in local optima, resulting in overfitting and limited performance. To overcome these limitations, the proposed model uses the Memory-Aware Fractional optimisation algorithm based on fractional calculus theory to eliminate premature convergence and prevent local optima while maintaining minimum loss. Here, the Sand Cat Swarm Optimisation (SCSO), Serval Optimisation Algorithm (SOA), and Improved SCSO are evaluated against MemAFO over 100 epochs based on the loss value. With 50 epochs, the MemAFO algorithm attains only 1.36 × 10^−34^ loss, whereas the existing SCSO and SAO exhibit losses of 0.058 and 0.145, respectively. When the number of epochs increases to 94, the loss generated by the MemAFO algorithm decreases to 9.94 × 10^−56^. At the same time, traditional SCSO and SAO exhibit high losses of 0.005 and 0.010, respectively. Thus, the proposed MemAFO algorithm shows minimal loss compared with existing SCSO and SOA optimisation techniques and ensures high performance in atherosclerosis classification. Moreover, the MemAF optimisation is integrated with the StyleGAN to mitigate overfitting and improve generalisability in the proposed MemAF-KTCBM model.

### 4.8 Statistical analysis

Statistical analysis provides a better interpretation of risk factors, establishing a reliable and transparent basis for making quantified decisions in atherosclerosis classification. For accurate and appropriate analysis, distinct statistical measures, such as the best, mean, variance, and standard deviation of accuracy, precision, NPV, recall, PPV, and MCC, are analysed. The statistical analysis of the MemAF-KTCBM model using the ARCADE and CADICA datasets is presented in Tables 5, 6.

**Table 5:** Statistical analysis of the proposed MemAF-KTCBM model with existing systems on the ARCADE dataset.

Analysis/methods		UNet	RFDS	3D-CNN	DenseNet-201	SCSO-KMTCN	SOA-KMTCN	MemAF-KTCBM (proposed)
Best	Accuracy	96.51	93.71	96.33	96.65	97.81	97.88	98.64
	MCC	0.91	0.94	0.94	0.94	0.95	0.95	0.96
	Recall	96.73	93.48	94.08	94.33	96.47	96.64	97.82
	NPV	0.96	0.94	0.94	0.95	0.97	0.97	0.98
	Precision	96.40	93.83	97.45	97.80	98.49	98.49	99.06
	PPV	0.96	0.94	0.97	0.98	0.98	0.98	0.99
Mean	MCC	0.88	0.89	0.89	0.90	0.91	0.91	0.93
	Precision	92.75	89.74	91.33	92.65	94.61	95.17	96.26
	PPV	0.93	0.90	0.91	0.93	0.95	0.95	0.96
	Recall	94.40	90.11	90.71	91.11	92.18	92.92	96.04
	NPV	0.93	0.90	0.91	0.91	0.92	0.93	0.94
	Accuracy	93.30	89.86	91.12	92.14	93.80	94.42	96.19
Variance	Accuracy	5.27	5.32	8.63	11.39	11.30	9.48	5.93
	NPV	0.0014	0.0005	0.0006	0.0007	0.0012	0.0013	0.0010
	Precision	6.34	5.22	10.70	14.79	13.26	10.26	9.74
	MCC	0.0003	0.0011	0.0011	0.0011	0.0010	0.0010	0.0010
	Recall	3.58	5.77	5.65	6.52	8.59	8.40	1.32
	PPV	0.0006	0.0005	0.0011	0.0015	0.0013	0.0010	0.0010
Standard deviation	Accuracy	2.30	2.31	2.94	3.37	3.36	3.08	2.43
	PPV	0.03	0.02	0.03	0.04	0.04	0.03	0.03
	Recall	1.89	2.40	2.38	2.55	2.93	2.90	1.15
	MCC	0.02	0.03	0.03	0.03	0.03	0.03	0.03
	Precision	2.52	2.29	3.27	3.85	3.64	3.20	3.12
	NPV	0.04	0.02	0.03	0.03	0.03	0.04	0.03

**Table 6:** Statistical performance of the proposed MemAF-KTCBM model with existing systems on the CADICA dataset.

Analysis/methods		UNet	RFDS	3D-CNN	DenseNet-201	SCSO-KMTCN	SOA-KMTCN	MemAF-KTCBM (proposed)
Best	Accuracy	95.25	92.49	95.12	95.50	96.70	96.77	97.73
	MCC	0.90	0.93	0.93	0.93	0.94	0.94	0.95
	Precision	95.14	92.61	96.23	96.64	97.36	97.38	98.14
	NPV	0.95	0.93	0.93	0.94	0.96	0.96	0.97
	Recall	95.47	92.26	92.90	93.21	95.37	95.55	96.91
	PPV	0.95	0.93	0.96	0.97	0.97	0.97	0.98
	NPV	0.91	0.88	0.89	0.90	0.91	0.91	0.93
	Accuracy	91.67	88.32	89.60	90.65	92.34	92.99	95.13
	MCC	0.87	0.87	0.88	0.88	0.89	0.90	0.92
	Precision	91.13	88.20	89.80	91.15	93.14	93.72	95.20
	PPV	0.91	0.88	0.90	0.91	0.93	0.94	0.95
	Recall	92.75	88.56	89.19	89.64	90.75	91.51	94.98
Variance	Accuracy	6.08	6.18	9.78	12.82	12.70	10.76	6.74
	Precision	7.21	6.08	11.93	16.34	14.69	11.54	10.72
	Recall	4.24	6.63	6.63	7.67	9.90	9.67	1.72
	MCC	0.0004	0.0012	0.0012	0.0012	0.0012	0.0011	0.0010
	NPV	0.0015	0.0006	0.0007	0.0008	0.0013	0.0014	0.0010
	PPV	0.0007	0.0006	0.0012	0.0016	0.0015	0.0012	0.0011
Standard deviation	Accuracy	2.47	2.49	3.13	3.58	3.56	3.28	2.60
	Precision	2.69	2.47	3.45	4.04	3.83	3.40	3.27
	Recall	2.06	2.58	2.58	2.77	3.15	3.11	1.31
	MCC	0.02	0.03	0.03	0.03	0.03	0.03	0.03
	NPV	0.04	0.02	0.03	0.03	0.04	0.04	0.03
	PPV	0.03	0.02	0.03	0.04	0.04	0.03	0.03

### 4.9 Sensitivity analysis

**Figure 13:**
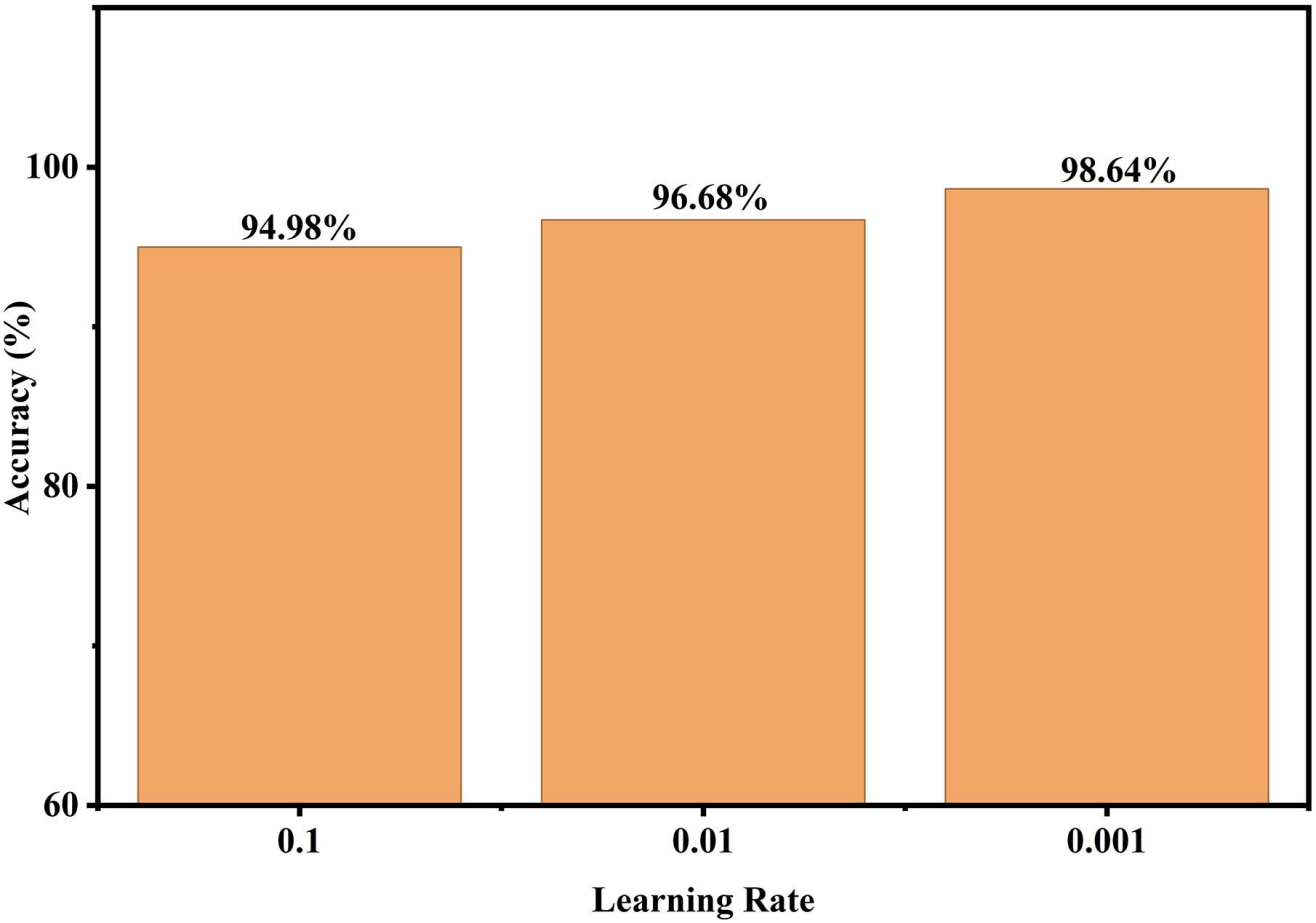
Sensitivity analysis of the proposed MemAF-KTCBM model.

Figure 13 presents the sensitivity analysis of the proposed model for multiple learning rates. During the atherosclerosis classification performance evaluation, the sensitivity of the proposed framework to the hyperparameters of the Memory-Aware Fractional Optimisation (MemAFO) strategy was evaluated using various learning rates: 0.1, 0.01, and 0.001. The proposed MemAF-KTCBM model achieved classification accuracies of 94.98%, 96.68%, and 98.64% for learning rates of 0.1, 0.01, and 0.001, respectively, demonstrating that the framework is less sensitive to the selected hyperparameters, with the learning rate of 0.001 providing the best performance. The integration of the MemAFO strategy tunes the model parameters to achieve a high convergence rate while reducing false errors.

### 4.10 Ablation study of model components

**Figure 14:**
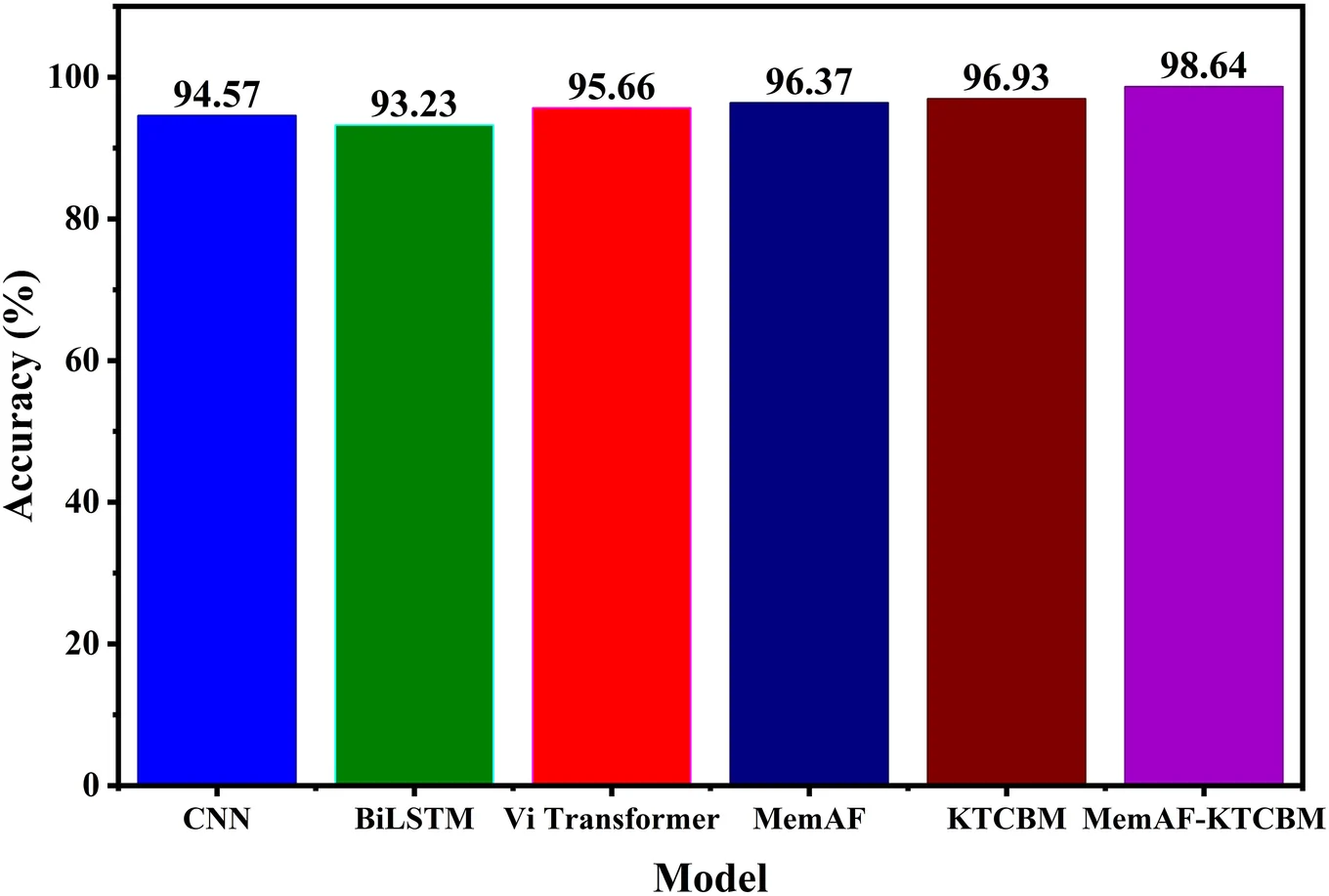
Ablation study results for the classification accuracy of the proposed MemAF-KTCBM model and its individual components, namely, CNN, BiLSTM, vision transformer (ViT), MemAF, and KTCBM.

Figure 14 presents the ablation study of the several components used in the proposed MemAF-KTCBM model based on classification accuracy. An ablation study is performed to justify the contribution of individual components, such as CNN, BiLSTM, ViT, MemAFO, and KTCBM, compared with the proposed MemAF-KTCBM framework to validate the atherosclerosis classification performance. As per the ablation study, the CNN model, the BiLSTM model, the vision transformer, and KTCBM achieved accuracies of 94.57%, 93.23%, 95.66%, and 96.98%, respectively. In addition, the MemAF optimisation fine-tunes the model parameters to achieve a high accuracy of 96.37%. Without the MemAF algorithm, the model exhibited lower performance and poor convergence during the classification process. Consequently, the hybrid MemAF-KTCBM model achieved a high accuracy of 98.64% by incorporating the strengths of all individual components to learn intricate features and predict disease uncertainties while maintaining high classification performance.

#### 4.10.1 Ablation study of MemAF-SGAN

**Figure 15:**
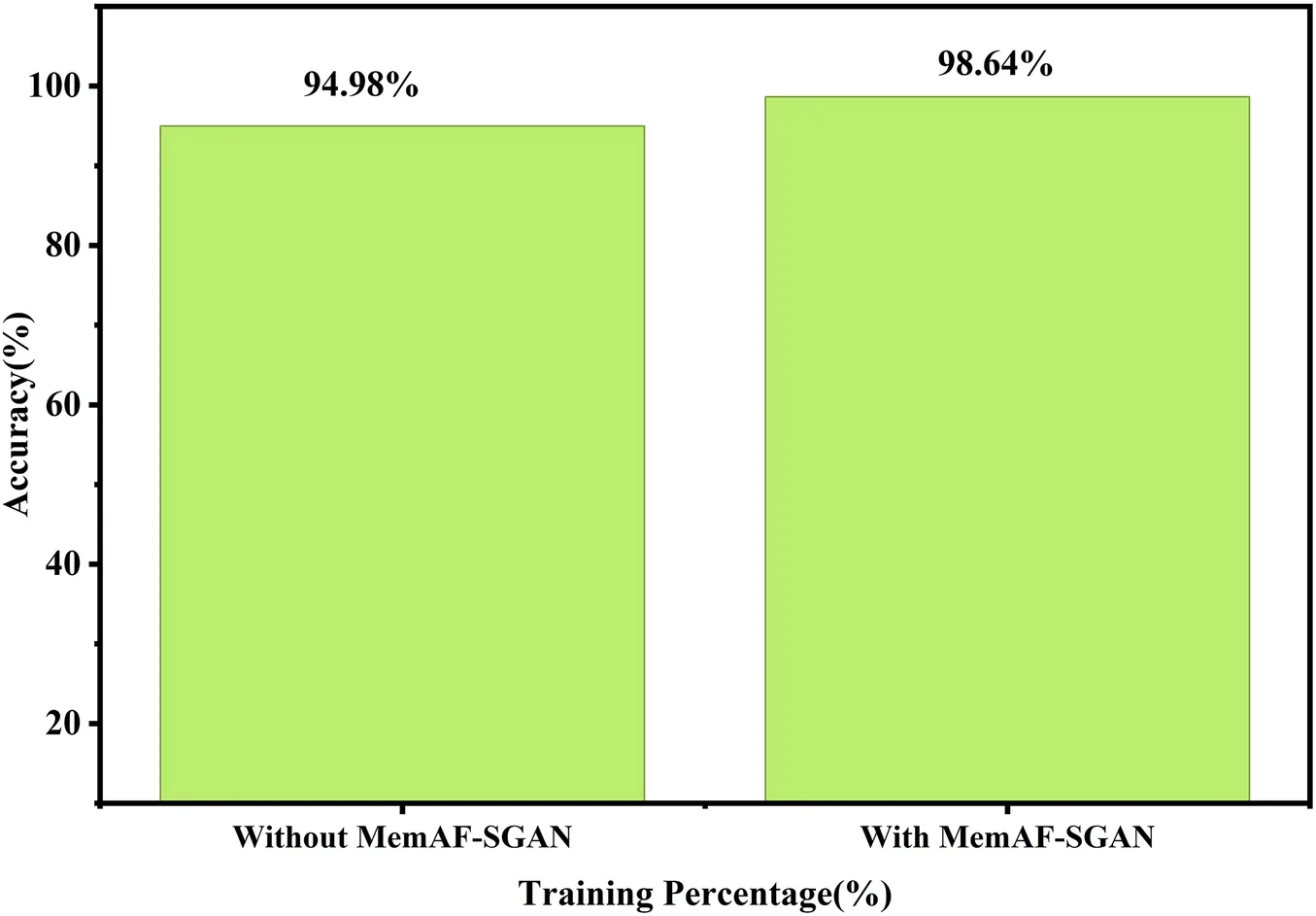
Ablation study of classification accuracy with and without MemAF-SGAN.

Figure 15 illustrates a comparison of the augmentation performance of the MemAF-KTCBM model with and without MemAF-SGAN integration based on accuracy values. As per the analysis, the proposed MemAF-SGAN-based augmentation model achieved an accuracy of 98.64%, compared to 94.98% without it. Moreover, the model incorporates MemAF optimisation with the StyleGAN model to boost performance and generate high-resolution synthetic images, effectively eliminating data imbalance and overfitting problems.

#### 4.10.2 Ablation study of MPSPNet-based segmentation

**Figure 16:**
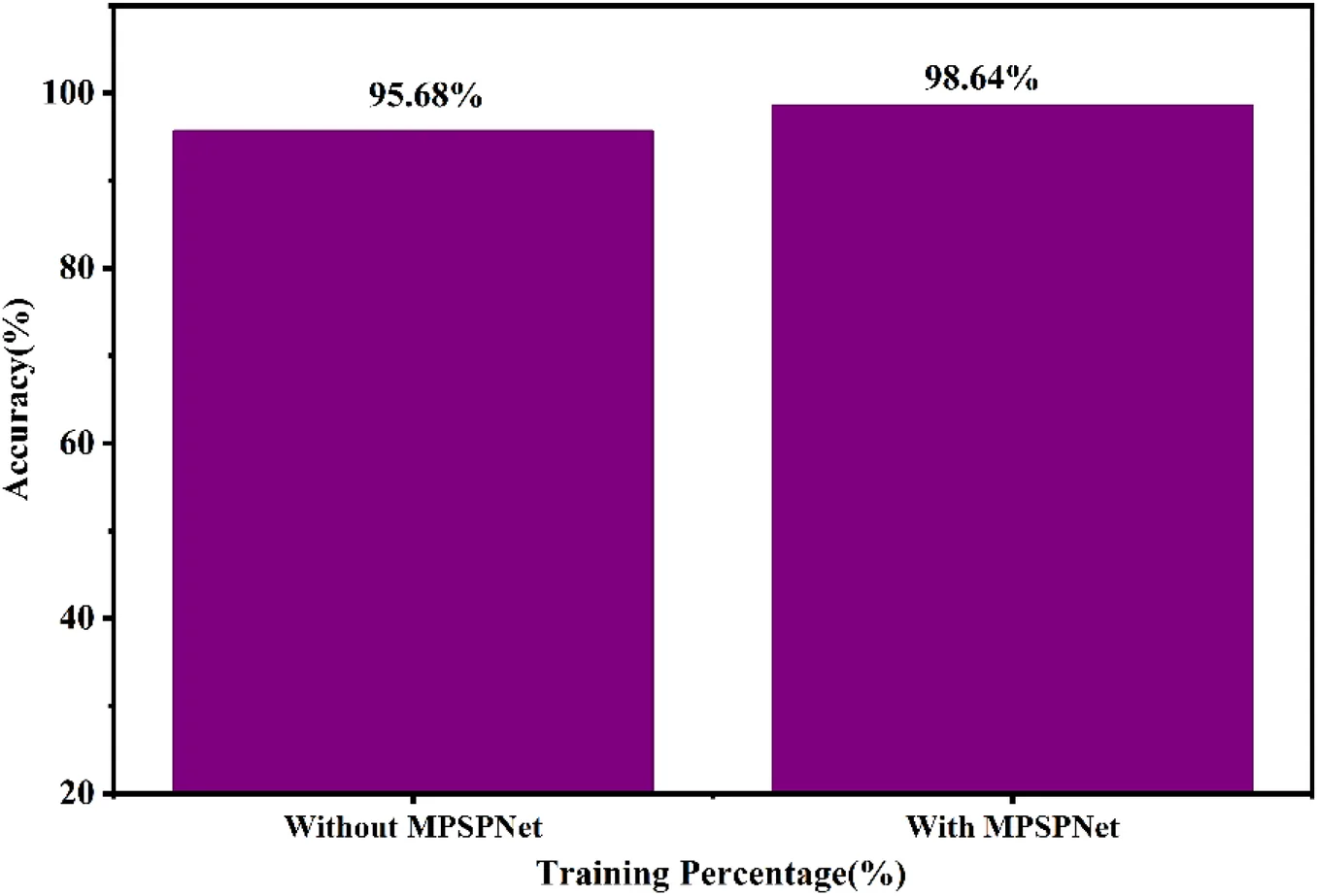
Ablation study of classification accuracy with and without MPSPNet-based segmentation.

Figure 16 presents a comparison of the segmentation performance of the MemAF-KTCBM model with and without MPSPNet integration based on accuracy values. As per the analysis, the proposed MPSPNet-based segmentation achieved an accuracy of 98.64%, compared with 95.68% without it. Moreover, the incorporation of MemAF optimisation with the pyramid pooling module extracts intricate features and enhances atherosclerosis classification performance while reducing errors.

### 4.11 Computational complexity analysis

**Figure 17:**
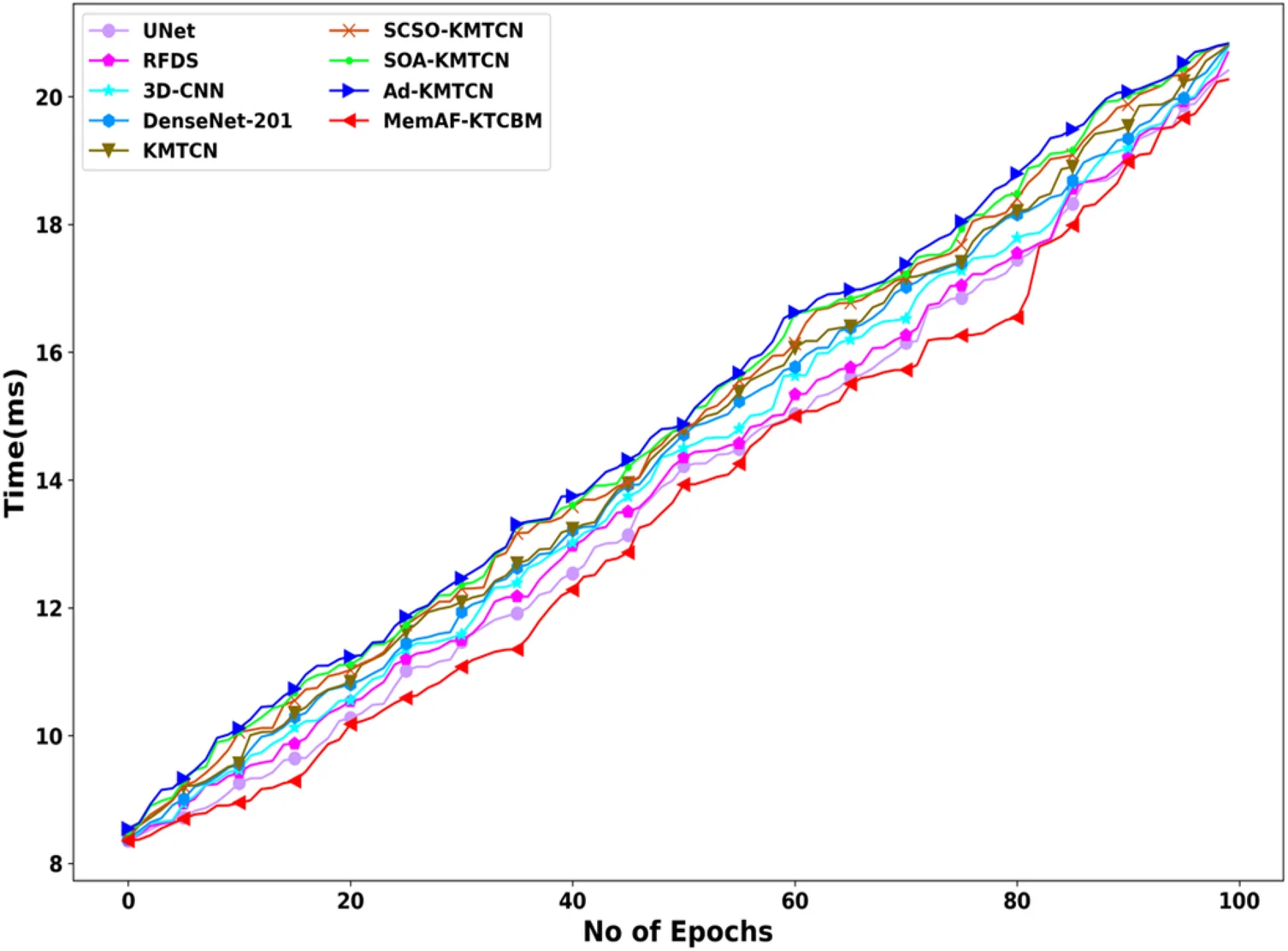
Computational complexity analysis.

The computation time of the MemAF-KTCBM model for atherosclerosis classification is compared with those of other existing methods across multiple iterations. The computational complexity analysis indicates that the MemAF-KTCBM model achieved a lower computation time of 20.27 ms and outperformed other approaches at the 100th epoch. The existing models incurred high computation times, including UNet (20.41 ms), RFDS (20.69 ms), 3D-CNN (20.78 ms), DenseNet-201 (20.80 ms), KMTCN (20.80 ms), SCSO-KMTCN (20.81 ms), SOA-KMTCN (20.82 ms), and Ad-KMTCN (20.83 ms). Specifically, the utilisation of MemAF optimisation with knowledge distillation tunes the model parameters to achieve high classification accuracy and enhances convergence speed while reducing computation complexity. Furthermore, the computational time complexity of the MemAF-KTCBM model is depicted in Figure 17.

### 4.12 Evaluation loss curve analysis

**Figure 18:**
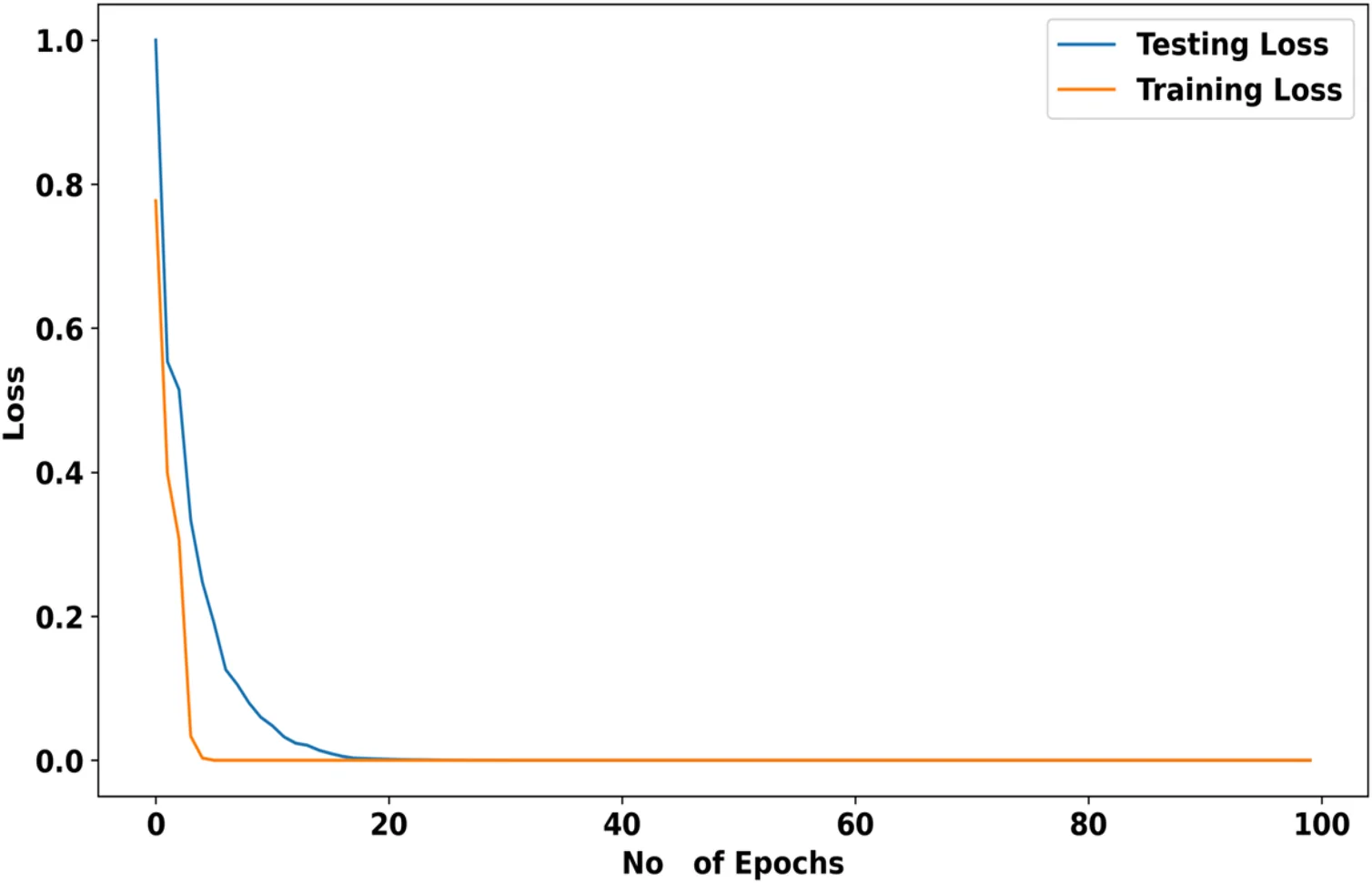
Evaluation loss curve analysis.

Figure 18 shows the evaluation loss curve of the MemAF-KTCBM model based on training and test losses for 100 epochs during atherosclerosis classification. At the initial epoch, the model achieved a test loss of 1 and a training loss of 0.78, which gradually decreased with increasing epochs. Moreover, the proposed model achieved a very low training loss of 0 and a test loss of $9.03\times 10^{-9}$ at the 98th epoch. Increasing the maximum number of epochs reduces loss and improves plant classification performance. Moreover, the integration of MemAF optimisation with the knowledge distillation mechanism increases the performance of the model and reduces loss.

### 4.13 Interpretability analysis

The interpretability of the MemAF-KTCBM model regarding atherosclerosis classification performance is demonstrated through the involvement of multiple architectures, such as CNN, BiLSTM, ViT, and KTCBM, within the proposed model, allowing the framework to learn complex patterns from input medical images. The MemAFO algorithm tunes the model parameters to increase the convergence rate and achieve high classification accuracy. The advanced MemAF-KTCBM model accurately identifies atherosclerosis classes with a 98% success rate and achieves a data exchange time of 112 ms. These results confirm that the proposed model provides high interpretability by extracting complex relationships from input medical images and improving overall success.

### 4.14 Confusion matrix

**Figure 19:**
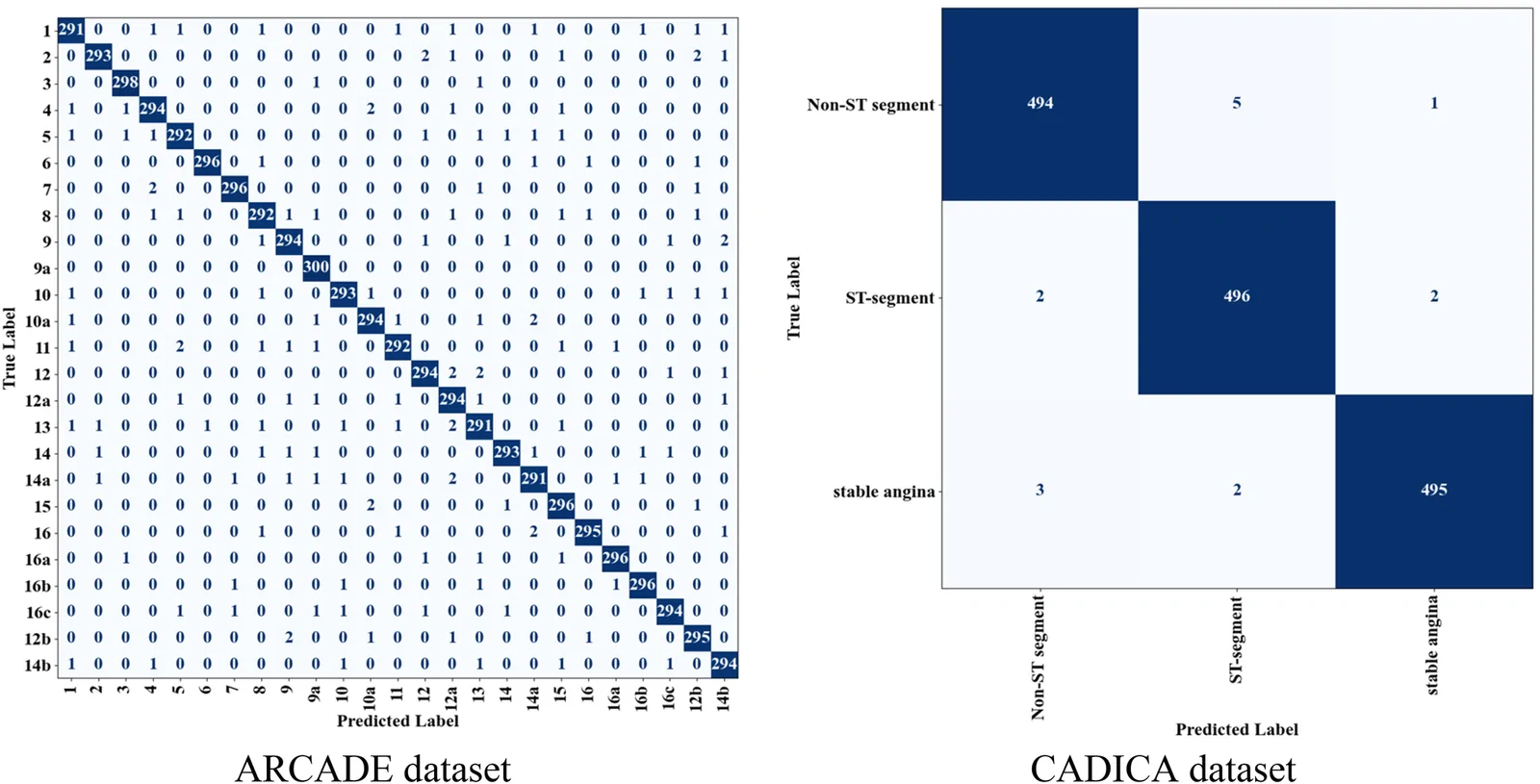
Confusion matrix.

Figure 19 depicts the confusion matrix of the proposed MemAF-KTCBM model for atherosclerosis classification using the ARCADE and CADICA datasets. The confusion matrix compares the original labels with the predicted labels, and correct predictions of atherosclerosis classes are visualised diagonally. According to the confusion matrix, the model correctly classified atherosclerosis into 25 classes for the ARCADE dataset, with 291 as class-1, 293 as class-2, 298 as class-3, and vice versa. For the CADICA dataset, the MemAF-KTCBM model correctly predicted three classes: 494 as non-ST-segment, 496 as ST-segment, and 495 as stable angina. Consequently, the model incorrectly predicted five non-ST-segment classes as ST-segment, two ST-segment classes as stable angina, and two stable angina classes as ST-segment, which is very low compared with the correct prediction classes, demonstrating high classification performance and accuracy.

### 4.15 Comparative discussion

Tables 7, 8 compare the atherosclerosis classification performance of the MemAF-KTCBM model with those of the traditional approaches using the ARCADE and CADICA datasets. Using the ARCADE dataset, the MemAF-KTCBM achieved 98.64% accuracy, 99.06% precision, 97.82% recall, 0.98NPV, 0.99PPV, and 0.96MCC at a 90% training split. The intensely deeper layers of the MemAF-KTCBM model, with knowledge distillation characteristics, accurately examine the input medical image features while reducing resource requirements (1). Eventually, the ViT present in the architecture with MHA simulates the MemAF-KTCBM model to focus on atherosclerotic features. The reduced generalisability issues (7) faced using traditional hybrid polynomial ensemble model (2) are effectively eliminated by generating high-quality images with characteristics similar to those of the input using the MemAF-SGAN. Thus, the MemAF-SGAN alleviates class imbalance and overfitting issues (4) by producing an optimally reliable atherosclerosis classification performance. Moreover, segmenting cardiac structures with MPSPNet ensures reliable atherosclerosis classification performance through its multi-scale feature aggregation. Specifically, the additional resource requirements associated with conventional approaches are reduced by extracting the best possible features using the pretrained local descriptor.

**Table 7:** Metricwise comparative discussion using the ARCADE dataset with 90% training and 10-fold cross-validation.

Methods/metrics	Accuracy (%)	Precision (%)	Recall (%)	NPV	PPV	MCC
Training percentage-90						
UNet (25)	96.51	96.40	96.73	0.96	0.96	0.91
3D-CNN (27)	96.33	97.45	94.08	0.94	0.97	0.94
SCSO-KMTCN (24)	97.81	98.49	96.47	0.97	0.98	0.95
DenseNet-201 (28)	96.65	97.80	94.33	0.95	0.98	0.94
SOA-KMTCN (23)	97.88	98.49	96.64	0.97	0.98	0.95
RFDS (26)	93.71	93.83	93.48	0.94	0.94	0.94
MemAF-KTCBM	**98** **.** **64**	**99** **.** **06**	**97** **.** **82**	**0** **.** **98**	**0** **.** **99**	**0** **.** **96**
10-fold						
UNet (25)	95.60	95.36	96.06	0.93	0.95	0.94
3D-CNN (27)	95.57	95.67	95.36	0.93	0.96	0.90
SOA-KMTCN (23)	96.83	97.06	96.36	0.96	0.97	0.94
DenseNet-201 (28)	95.82	95.74	95.99	0.95	0.96	0.93
SCSO-KMTCN (24)	96.32	96.37	96.21	0.95	0.96	0.94
RFDS (26)	95.47	95.54	95.32	0.90	0.96	0.89
MemAF-KTCBM	**97** **.** **53**	**97** **.** **82**	**96** **.** **94**	**0** **.** **97**	**0** **.** **98**	**0** **.** **95**

Bold values represent the best-performing results for each metric obtained by the proposed MemAF-KTCBM model.

**Table 8:** Metricwise comparative discussion using the CADICA dataset with 90% training and 10-fold cross-validation.

Methods/metrics	Accuracy (%)	Precision (%)	Recall (%)	NPV	PPV	MCC
Training percentage-90						
UNet (25)	95.25	96.40	95.47	0.95	0.95	0.90
SCSO-KMTCN (24)	96.70	98.49	95.37	0.96	0.97	0.94
3D-CNN (27)	95.12	97.45	92.90	0.93	0.96	0.93
RFDS (26)	92.49	93.83	92.26	0.93	0.93	0.93
SOA-KMTCN (23)	96.77	98.49	95.55	0.96	0.97	0.94
DenseNet-201 (28)	95.50	97.80	93.21	0.94	0.97	0.93
MemAF-KTCBM	**97** **.** **73**	**99** **.** **06**	**96** **.** **91**	**0** **.** **97**	**0** **.** **98**	**0** **.** **95**
10-fold						
UNet (25)	94.11	93.88	94.56	0.92	0.94	0.93
3D-CNN (27)	94.40	94.50	94.20	0.92	0.94	0.89
DenseNet-201 (28)	94.68	94.60	94.85	0.94	0.95	0.92
SCSO-KMTCN (24)	95.23	95.28	95.12	0.94	0.95	0.93
RFDS (26)	94.19	94.26	94.04	0.89	0.94	0.88
SOA-KMTCN (23)	95.76	95.99	95.29	0.95	0.96	0.93
MemAF-KTCBM	**96** **.** **65**	**96** **.** **94**	**96** **.** **07**	**0** **.** **97**	**0** **.** **97**	**0** **.** **94**

Bold values represent the best-performing results for each metric obtained by the proposed MemAF-KTCBM model.

## 5 Conclusion

The proposed methodology utilises an effective MemAF-KTCBM framework for accurate atherosclerosis classification using x-ray angiographic images. Effective tuning of the MemAF-KTCBM parameters using the MemAF optimisation algorithm eliminates premature convergence issues and attains improved model stability. Furthermore, the generation of high-quality, synthetic images using MemAF-SGAN mitigates class imbalance while ensuring better generalisability to diverse inputs. Fortunately, the use of the pretrained local descriptor to extract the best possible features from the MPSPNet-segmented cardiac structure reduced the additional resource requirements for atherosclerosis classification and simplified the process. Eventually, these special features enhanced the interpretability of the atherosclerosis classification process and improved overall performance. In particular, the deeper infrastructure of the MemAF-KTCBM model, combined with the ViT, facilitated the effective processing of crucial features extracted from the high-quality preprocessed images. Experimental results validated the atherosclerosis classification performance of the MemAF-KTCBM model, achieving 98.64% accuracy, 99.06% precision, 97.82% recall, 0.98 NPV, 0.99 PPV, and 0.96MCC at a 90% training split on the ARCADE dataset. In the future, eXplainable Artificial Intelligence techniques will be incorporated into the MemAF-KTCBM model to enhance decision-making and improve the accuracy of atherosclerosis classification. The proposed model can be further improved by incorporating additional datasets to enhance interpretability and generalisability.

## Data Availability

The original contributions presented in the study are included in the article/Supplementary Material; further inquiries can be directed to the corresponding author.

## Glossary

KTCBM: Knowledge-distilled Mutual-conditioned dynamic Transformer-enabled Convolutional neural network-Bidirectional long short-term Memory
MemAF: Memory-Aware Fractional optimisation
CNN: convolutional neural network
BiLSTM: bidirectional long short-term memory
MPSPNet: Memory-aware fractional optimisation-enabled Modified Pyramid Scene Parsing Network
MemAF-SGAN: Memory-Aware Fractional optimisation-enabled Style Generative Adversarial Network
CVD: cardiovascular disease
MRI: magnetic resonance imaging
OCT: optical coherence tomography
CCTA: coronary computed tomography angiography
IVUS: intravascular ultrasound
DL: deep learning
ML: machine learning
DBN: deep belief network
RNN: recurrent neural network
LSTM: long short-term memory
MACAN: multi-stream attention-guided coronary analysis network
SAM-VM-UNet: segment anything model-Vision Mamba-UNet
HPEM: hybrid polynomial ensemble model
CIDN: context-interactive deep network
DFA-Net: dual multi-scale feature aggregation network
QPSO: Quantum-behaved Particle Swarm Optimisation
SVM: support vector machine
AdaIN: Adaptive Instance Normalisation
RGB: red green blue
ReLU: rectified linear unit
LFD: local frequency descriptor
VGG-16: Visual Geometry Group 16
CAM: class activation map
MBConv: Mobile inverted Bottleneck Convolution
ViT: vision transformer
MLP: multi-layer perceptron
MHA: multi-headed attention
GELU: Gaussian error linear unit
OS: operating system
RAM: random-access memory
ROM: read-only memory
ARCADE: automatic region-based coronary artery disease diagnostics using x-ray angiographic images
CADICA: coronary artery disease detection using the invasive coronary angiography
NPV: negative predictive value
MCC: Matthews correlation coefficient
PPV: positive predictive value
RFDS: redundancy feature diminishment strategy
3D-CNN: three-dimensional convolutional neural network
DenseNet: dense network
SCSO: Sand Cat Swarm Optimisation
SOA: Serval Optimisation Algorithm
ISCSO: Improved Sand Cat Swarm Optimisation
XAI: eXplainable Artificial Intelligence
